# Explaining the dependence of M-site diffusion in forsterite on silica activity: a density functional theory approach

**DOI:** 10.1007/s00269-020-01123-5

**Published:** 2020-11-24

**Authors:** Joshua M. R. Muir, Michael Jollands, Feiwu Zhang, Andrew M. Walker

**Affiliations:** 1 Institute of Geochemistry, Chinese Academy of Sciences, 99 West Lincheng Road, Guiyang, 550081 Guizhou China; 2 School of Earth and Environment, University of Leeds, Leeds, LS2 9JT UK; 3 Institute of Earth Sciences, University of Lausanne, 1015 Lausanne, Switzerland; 4 Lamont-Doherty Earth Observatory, 61 Rt 9W, Palisades, NY 10964 USA

**Keywords:** Forsterite, Mg diffusion, DFT, Point defects, Enstatite, Aluminium

## Abstract

Experimentally, silica activity (aSiO_2_) has been shown to have an effect on Mg diffusion in forsterite, but no fully satisfactory mechanism has yet been proposed. We calculated the effects of aSiO_2_ and aluminium content (the main contaminant in some recent experimental studies), and their co-effect, on Mg diffusion in forsterite, using thermodynamic minimisations of defect formation energies [calculated using density functional theory (DFT)] and a Monte-Carlo diffusion model. These two variables, in isolation, do not appreciably change the defect concentrations of forsterite and thus do not affect the diffusivity of Mg. However, when elevated together, they cause large increases in the Mg vacancy content and thus can increase the Mg diffusivity by one to six orders of magnitude depending on temperature, with little pressure dependence. This effect is largely independent of Al_2_O_3_ concentration above ~ 1 wt. ppm, and thus, for all practical purposes, should occur wherever forsterite is in the presence of enstatite. It is also largely dependent upon configurational entropy and is thus highly sensitive to the chemistry of the crystal. A low concentration of structurally bound hydroxyl groups at low temperatures (1000 K) suppresses this effect in pure forsterite, but it is likely robust in the presence of water either when alternative water sinks (such as Ti or Fe) are present, or at high temperatures (> 1500 K). This effect is also robust in the presence of ferrous iron (or other substitutional Mg defects) at all temperatures. Fe_2_O_3_ can operate like Al_2_O_3_ in this reaction and should enhance its effect. These findings explain the experimentally observed dependency of Mg diffusion of aSiO_2_, and elucidate how chemical activity variations in both experiments and natural settings could affect not only the diffusivity of Mg in forsterite, but of olivine-hosted cations in general.

**Electronic supplementary material:**

The online version of this article (10.1007/s00269-020-01123-5) contains supplementary material, which is available to authorized users.

## Introduction

Understanding Mg diffusion in olivine is important for considerations of various geochemical transport processes including electrical conductivity (Fei et al. 2018), rheology (Jaoul 1990) and resolving the timescales of volcanic process (Costa et al. 2008), as well as for the understanding of point defect chemistry in silicates in general (Dohmen and Chakraborty 2007; Nakamura and Schmalzried 1983). Forsterite, the magnesian end member of olivine (Mg_2_SiO_4_), can exist in the pure Mg-Si–O system along with either periclase (MgO) or enstatite (MgSiO_3_). Whilst it has been understood for several decades that the buffering assemblage, and hence the silica activity, affects various properties of olivine such as its rheology (Bai et al. 1991; Ricoult and Kohlstedt 1985), trace element incorporation [e.g. H (Matveev et al. 2001)] and its point defect population (Nakamura and Schmalzried, 1983; Stocker and Smyth, 1978), the extent to which aSiO_2_ affects trace, minor and major element diffusion in olivine is still being elucidated.

Specifically, it has been recently demonstrated that various M-site cations, including Ni^2+^,Co^2+^ (Zhukova et al. 2014); Cr^2+^_,_ Cr ^3+^ (Jollands et al. 2018); Zr^4+^, Hf^4+^ (Jollands et al. 2014); Mn^2+^, Ni^2+^, Co^2+^, Ti^3+^, Ti^4+^ (Jollands et al. 2016b); Rh^3+^ (Zhukova et al. 2018) and Al^3+^ (Zhukova et al. 2017), all show higher diffusivities (0.5–2 orders of magnitude) in nominally-pure forsterite buffered with enstatite compared to equivalent experiments buffered with periclase. Only Be^2+^ (Jollands et al. 2016a), Ca^2+^ (Bloch et al. 2019) and H^+^ (Jollands et al. 2016c) have yet shown diffusivities independent of aSiO_2_. Recently, Mg^2+^ diffusivity has been shown to be ~ 1 order of magnitude higher in enstatite versus periclase-buffered experimental assemblages (Jollands et al. 2020). It should be noted that some previous experimental campaigns showed no effect of aSiO_2_ on Mg diffusion ()—Chakraborty et al. 1994; Andersson et al. 1989this has been proposed to relate to the inherent difficulty of controlling aSiO_2_ in such experiments (Jollands et al. 2020). Additionally, previous theoretical studies (Walker et al. 2009; Bejina et al. 2009) have not considered the effect of aSiO_2_ on diffusion. Previous attempts (e.g. Zhukova et al. (2014)) to link the experimentally observed aSiO_2_—diffusivity relationship to the point defect population of forsterite have relied on the papers of Stocker and Smyth (1978), Pluschkell and Engell (1968), Smyth and Stocker (1975). These studies calculated the charge balance conditions of a set of defect-producing reactions, based on how variations of the defect concentrations would affect the equilibrium conditions. These works, however, involved major assumptions due to a lack of quantification of the energetics of these reactions. In these studies, it was assumed that changing aSiO_2_ causes a change in Mg vacancy concentration that can be described by a simple exponent, which is unlikely when considering all of the Mg vacancies that are formed by all the defect reactions together.

To address this, we use density functional theory (DFT) (Hohenberg and Kohn 1964; Kohn and Sham 1965) to examine the concentration at thermodynamic equilibrium of point defects in forsterite, and how this value is changed by the presence of enstatite or periclase (i.e. changing the aSiO_2_). We also investigate the effect of small concentrations of Al, the only notable impurity in the crystals used in most of the experiments where an effect of aSiO_2_ was demonstrated (Zhukova et al. 2014; Jollands et al. 2020). Whilst the results are only applicable to pure forsterite, we also use thermodynamics to speculate how Mg diffusion would operate in natural settings.

## Methods

Three steps were necessary to form a complete picture of Mg diffusion in forsterite. First, the number of defects present in forsterite was determined—for this, the energetics of the defect formation reactions are needed. Thus, the energies of all the plausible defects forming reactions were calculated, both with and without enstatite, at various pressures and temperatures. This was done using lattice dynamics, with the force constants and energies provided by DFT calculations. This provides the energy of a series of isolated reactions. In real forsterite, the defect producing reactions will interact with each other. Therefore, in the second step, the energies of the reactions calculated in step one were used to build a thermodynamic model. With this model, the concentration of defects at thermodynamic equilibration can be found through a free energy minimization, so that the number of defects present in forsterite under varying conditions can be obtained. Third, the effects of these defect concentrations on diffusion were determined. For this, the defect concentrations established from the first two steps were converted into diffusivities by inserting them into an Mg diffusion kinetic Monte Carlo model.

### Reaction free energies

Free energies were calculated with the CASTEP code (Clark et al. 2005). This solves for the electronic structure (and thus the energy) of any structure using DFT with a plane-wave basis set for the valence electrons and pseudopotentials for the core electrons and nuclei. An approximation for the exchange–correlation functional is needed, for which we used the Perdew–Burke–Elmer (PBE) approximation (Perdew et al. 1997, 1998). Ultrasoft pseudopotentials (Payne et al. 1992) were generated using the on-the-fly method of CASTEP 16.11 in the PBE scheme—the valence shells are: Al: 3 s and 3p**;** Mg: 2 s, 3p and 3 s; O: 2 s and 2p; Si: 3 s and 3p. To find minimum energy structures, we used the standard quasi-Newtonian minimization routine (Pfrommer et al. 1997) implemented in CASTEP.

The energies of all (likely) relevant defects (Mg, Si and O interstitials and vacancies, and Al on Mg and Si sites) were calculated by placing them into a forsterite (2 × 1 × 2) supercell. The energies of forsterite (2 × 1 × 2 cell), MgO (2 × 2 × 2) and enstatite (1 × 1 × 2) supercells were also calculated. Enstatite energies were calculated in its ortho, proto and clino forms, and the lowest energy phase used at any particular point in *P*,T space. All structures were relaxed with a plane wave cutoff of 1000 eV, 4 × 4 × 4 k-points and relaxed to a force tolerance of 0.01 eV/Å and an energy tolerance of 1 × 10^–5^ eV/atom, although for frequency calculations this was increased to 0.001 and 1 × 10^–9^, with plane wave and k-point cutoffs set to levels where the tolerances were repeated across runs. Calculations were done at 0, 5, 10 and 15 GPa. For cells that had a formal charge, the energy calculated by CASTEP includes a defect–defect interaction term between adjacent supercells which does not reflect our desired energy of a point charge in an infinite medium. We can approximately correct for this term, however, by assuming it is the energy of a periodic array of point charges in a uniform neutralizing background charge. This was done using the method of Leslie and Gillan (1985), first used for forsterite by Brodholt and Refson (2000)**.** To use this method, the relative permittivity of the cell needs to be set—we used a value of 6.2 (Weast 1981). The charge–charge interaction energy which is to be removed can then be found by calculating the energy of a periodic array of ions and dividing by the vacuum constant. To determine the energy of the periodic array we determined the Ewald sum with the GULP code (to ease calculation) (Gale 1997). This sum was determined for a forsterite (2 × 1 × 2) unit cell using the TBH1 force field (Price et al. 1987) (which is an array of point charges) and the Wolf sum approximation to the Ewald sum with *R*_cut_ of 15 Å. Full details of this force field are given in Muir et al. (Submitted).

In all simulations, we have a fixed concentration of defects, but we intend to find the effect of introducing a defect at the “dilute limit”. This is where the concentration of defects is small enough that the defect–defect interaction terms are non-existent, defect–defect and charge–charge interactions do not depend upon the specific chemistry and relaxation around defects and the introduction of defects does not affect bulk parameters such as volume and permittivity. Simulating this point requires very large supercells which are computationally extremely demanding for little gain in accuracy. To approximate the dilute limit, we fixed the volume of all of our defect-containing supercells to the volume of a defect-free supercell to remove spurious unit-cell expansion terms which would not be present at the dilute limit. Similarly, when correcting for charge–charge interactions we always set the permittivity constant to that of a defect-free cell as at the dilute limit the introduction of defects should not change this constant. To test the inaccuracy caused by our supercells being too small, we calculated the defect energy of $V_{{{\text{Mg}}}}^{\prime \prime }$, ${V}_{\mathrm{O}}^{\bullet \bullet }$, ${\mathrm{Al}}_{\mathrm{Mg}}^{\bullet }$ and ${\mathrm{Al}}_{\mathrm{Mg}}^{\bullet }$ in their most stable positions in our normal (2 × 1 × 2) and an extra large (4 × 2 × 4) supercell and we find the difference in defect energy to be less than 30 meV/defect in all cases and for $V_{{{\text{Mg}}}}^{\prime \prime }$ to be less than 10 meV/defect.

To determine the free energy as a function of temperature, we calculated the phonon frequencies for all perfect and defected systems using the finite displacement method of CASTEP (Frank et al. 1995) with finite displacements of 0.01 bohr (0.00053 Å). All phonons were calculated at *q* = (0,0,0), as with finite displacement this is the only physically meaningful q-point. To calculate (for example) equations of state typically extremely sensitive force calculations using DFPT and multiple *q*-points are required, as the relevant parameter is the absolute value of each phonon. In our case, we require only the relative value of each phonon as we are calculating simply the difference between a perfect and defected supercell and thus less accuracy is required. While our single point calculation may introduce a significant sampling error in absolute terms, in relative terms it is likely to be small. Additionally, the effects of vibrational entropy are very small when compared to those of configurational entropy, as discussed below. Finally, we determine the effect of temperature on reactions with very large reaction energies and so the sub meV/atom accuracy of multiple *q*-point DFPT is not required. Therefore, additional *q*-points will not have any large effect upon the results as they change only the least significant term in the energy equation. To test these assumptions, we calculated the energy of a perfect forsterite unit cell and one containing a $V_{{{\text{Mg}}}}^{\prime \prime }$ using both the method outlined above and density phonon perturbation theory (as implemented in CASTEP) with q-points (2 × 2 × 2). We then calculated the difference in defect energy determined with these two methods and found it to be less than 10 meV/defect, which is much smaller than our reaction energies.

Frequencies were calculated for at least five different volumes and then the Gibbs free energy (*G*) at each volume (*V*), pressure (*P*) and temperature (*T*) was found with:

1 $$G\left( {P,T,V} \right) = U\left( V \right) + PV + E_{ZP} \left( V \right) - TS\left( {T,V} \right),$$

where *U* is the internal energy. *E*_*ZP*_ (zero point energy) and *S* (entropy) are calculated using Eqs. 2 and 3, respectively:

2 $$E_{ZP} \left( V \right) = \mathop \sum \limits_{k,i} \frac{1}{2}\hbar\nu_{k,i} \left( V \right),$$

3 $$S\left( {V,T} \right) = - \mathop \sum \limits_{k,i} \ln \left[ {1 - \exp \left( { - \frac{{ \hbar\nu_{k,i} \left( V \right)}}{{k_{B} T}}} \right)} \right] - \frac{1}{T}\mathop \sum \limits_{k,i} \hbar\nu_{k,i} \left( V \right)\left[ {\exp \left( {\frac{{ \hbar\nu_{k,i} \left( V \right)}}{{k_{B} T}}} \right) - 1} \right]^{ - 1} ,$$

where *ν*_*k,i*_ is the frequency of the phonon with wave vector *k* in the *i*-th band, *k*_*b*_ is the Boltzmann constant and ${\hbar }$ is the reduced Planck constant. At the pressure and temperature of interest, the appropriate volume and free energy were determined by fitting second-order polynomials across our volume range and minimizing Eq. 1. This method is quasi-harmonic as it includes thermal expansion of the crystal but ignores the effect of anharmonicity in phonon vibrations and thus fails when these are significant.

### Pressure correction

While DFT generally reliably reproduces pressure derivatives, the absolute pressures reported by DFT (*P*^DFT^) are known to be systematically incorrect, in that they are shifted in one direction. This arises due to the use of an approximation of the exchange–correlation term as different approximations give different pressure shifts. Generalized gradient approximation (GGA) methods (such as the PBE approximation used here) overestimate volume and pressure, whereas local density approximation (LDA) methods have the opposite effect. As pressure differences are reliably reported (as the effects of the approximation largely cancel out) replication of experimental elasticities has been performed via simple correction schemes based on experimental values. As shown in Zhang et al. (2013) for example simple linear correction schemes often produce sensible results, e.g.,:

4 $$P\left( {V,T} \right) = P^{{{\text{DFT}}}} \left( {V,T} \right) - P^{{{\text{DFT}}}} \left( {V_{0}^{\exp } } \right),$$

where ${V}_{0}^{\mathrm{exp}}$ is the experimental volume at 0 pressure determined from fitting to an equation of state. While such corrections are likely possible for defect energies, which are strong functions of pressure, no rigorously tested formulations have been produced due to the paucity of reliable experimental defect energy data. Regardless, we can consider the magnitude of this pressure correction effect by using Eq. 4, i.e. assuming defect energies are linear functions of DFT pressure. For this equation, we used ${V}_{0}^{\mathrm{exp}}$ values of 287.4 Å^3^ for olivine (Isaak et al. 1989), 74.71 Å for MgO (Speziale et al. 2001) and 832.918 Å^3^ for enstatite (Kung et al. 2004). This provided corrections of − 4.95, − 4.45 and − 3.91 GPa, respectively. As we are assuming a dilute limit, these pressure corrections are fixed regardless of the defects present in the unit cell. To simplify discussion, the values in this work have been corrected by − 5 GPa such that values are presented at 0, 5 and 10 GPa, but were calculated at a DFT pressure of 5, 10 and 15 GPa. This will provide pressure values closer to actuality while still being somewhat inaccurate. The pressure derivatives between these three runs at different pressures should be much more reliable.

### Defect sites

There are many different types of defects that can exist in forsterite. In this study, we focus on vacancies and interstitials of the major elements in forsterite as well as two substitutional sites of Al. Each of the defects were allowed to exist on the following sites in Kröger-Vink notation (Kroger and Vink 1956): $V_{{{\text{Mg}}}}^{\prime \prime }$: M1 and M2; ${V}_{\mathrm{O}}^{\bullet \bullet }$: O1, O2 and O3; $V_{{{\text{SI}}}}^{\prime \prime \prime \prime }$: Si; ${\mathrm{Mg}}_{I}^{\bullet \bullet }$: M1 and I2; ${\text{O}}_{I}^{\prime \prime }$ $:$ I1, I2, T1, T2, T3, T4 and T5; ${\mathrm{Si}}_{I}^{\bullet \bullet \bullet \bullet }$: I1, I2, T1, T2, T3, T4 and T5; ${Al}_{\mathrm{Si}}^{^{\prime}}:$ Si; ${Al}_{\mathrm{Mg}}^{\bullet }:$ M1 and M2, where I1 and I2 are vacant octahedral sites, T1–T5 are vacant tetrahedral sites, Si is the (unique) Si site, M1 and M2 are the two different Mg sites and O1–O3 are the three different O sites in forsterite. A picture of forsterite with some sites labelled is shown in Figure S1.

I1 and I2 sites are equivalent to M1 and M2 sites, respectively, that have been shifted by 0.5 of the [100] unit cell vector. T1–T5 can be related to the O sites. Each oxygen site has two tetrahedral sites that are shifted by ± 0.34 in the [100] unit cell vector. The difference between the positive-shifted and negative-shifted tetrahedral sites are dependent on the local O environment in its SiO_4_ tetrahedron rather than in absolute terms. Therefore, we define the sites by the relative location of the O from which the site is shifted, and the Si to which that O is bonded. So, sites T1, T3 and T4 are equivalent to the sites O1, O2 and O3, respectively, shifted 0.34 in the [100] unit cell vector away from Si, and sites T2 and T5 are equivalent to the sites O2 and O3, respectively, shifted 0.34 in the [100] unit cell vector towards Si. The site that occurs when O1 sites are shifted by 0.34 in the [100] unit cell vector towards Si is simply the usually occupied Si site.

Mg interstitials have unique geometry in forsterite, being able to occupy empty octahedral sites (I1 and I2) as well as occupied octahedral sites (M1 and M2). This occurs through creating a “split” interstitial whereby two Mg occupy a single octahedral site. The two Mg are shifted either positively or negatively, respectively, in the [010] direction such that they are on either side of the site centre (Walker et al. 2009; Jaoul et al. 1995, Muir et al. Submitted)**.** Mg interstitials could not be stabilized on M2 or I1 sites, leaving split M1 and I2 as the only possible sites for Mg interstitials.

### Thermodynamic minimization

In a system at equilibrium, the concentration of the different types of defects will be whichever collective configuration of defects gives the lowest free energy. Thus, to find the concentrations of defects we need to be able to calculate the free energy for arbitrary concentrations of defects. This is done by firstly defining a set of reactions (R1…R11 see text) such that for any given set of defect concentrations (arrangement), a set of constants (1D reaction vectors) (x_1_,x_2_…x_11_) are defined between 0 and 1. Thus, if reaction R1 is proceeded forward by x_1_, R2 by x_2_… R11 by x_11_, then the correct defect concentration will be obtained. It should be noted that the overall defect concentration is varied by R1…R11 and thus it is not set and changes until it reaches the defect concentration that corresponds to the thermodynamic minimum. There are some additional constraints on the constants such that negative quantities of defects or of MgO/MgSiO_3_/Mg_2_SiO_4_ are not created, but the values of the constants are so small in defect calculations that these constraints are not naturally violated. The energy of this arrangement is then determined as such:

5 $$G = U + PV - TS_{{{\text{Vib}}}} - TS_{{{\text{Config}}}}$$

where *U* is the internal energy, *T* is the temperature, *P* and *V* are pressure and volume (enthalpy *H* is equal to *U* + PV) and *S*_Vib_ and *S*_config_ are the vibrational and configurational entropies, respectively. The first two terms of Eq. 5 (the enthalpy and lattice vibration change) can be arrived at through Eq. 6:

6 $$U + PV - TS_{{{\text{Lattice}}}} = \mathop \sum \limits_{i = 1}^{11} x_{i} *G_{{{\text{reaction}}}},$$

where *G*_reaction_ is the calculated change in the free energy for each reaction at the appropriate *P* and *T* (as calculated by CASTEP) without considering the configurational entropy term. The last term in Eq. 5 represents the configurational entropy of the arrangement. This is complex in forsterite due to both the large number of defects that exist on Mg sites and their spread across four different sites (M1, M2, I1 and I2) and the fact that with typical vacancy concentrations there are an extremely large number of possible configurations. To solve for this, we need to use a number of assumptions and the Gibbs entropy formula. In summary, this this is done through tabulating all possible combinations of each defect type confined to one of its possible sites, calculating the energy of each of these combinations using the relative energy of each defect at its appropriate site, the number of configurations of each of these combinations using the Stirling approximation and then the configurational entropy of all configurations of all combinations using the Gibb’s entropy formula. A full breakdown and discussion of this calculation are presented in the supplementary methods.

A major assumption in this minimization is that all defects are randomly distributed across the crystal and do not associate with one another and therefore their defect–defect interaction terms are small. This is justified by the fact that typical configurational entropy gains associated with randomly distributing defects are much larger than the pairing energy gains associated with placing two oppositely charged defects close to each other, when there is a small number of defects. This assumption was tested by calculating the pairing energy of the major defect pairs (Table S1) and how many defects would be needed before the pairing energy became larger than the configurational entropy in a simple ideal system with no other defects. Generally, much larger concentrations than that seen in reality were needed before this term became significant and so we assumed all defects were freely mobile and not associated with their charge pair. This conclusion shows that minimizing the defect–defect interaction terms by separating the defects reduces the total energy.

In summary, each reaction proceeding to the right produces a set of defects, and then these defects are spread across their different sites according to the energy differences between these sites and the thermodynamic minimum of this distribution. This is determined by the state of every other reaction. An alternative way to consider or calculate this is that (for example) Reaction 1 consiste of four reactions—one producing an M1 vacancy and an M1 interstitial, one producing an M2 vacancy/M1 interstitial, one an M1 vacancy/I2 interstitial and one a I2 vacancy/M2 interstitial. However, this will produce the same answer as a single reaction producing a vacancy and an interstitial that are then thermodynamically distributed across their two sites including all the configurational entropy of the different distributions across the sites.

Obtaining thermodynamic minima from these equations is difficult as the energy surfaces are complex, with many local minima. Furthermore, defect concentrations (and thus x_1_⋯x_11_) can have relative concentrations that vary by 50 + orders of magnitude.

To account for this, we used a customized numerical minimization procedure. For each temperature and pressure of interest, the Al content was set to the highest considered concentration (750 wt. ppm) and enstatite was set to zero. Then a brute force examination was run, varying all *x* values between 1 × 10^–20^ and 1 with points spaced half an order of magnitude apart for an estimate of the range of each of the values. The 5000 most stable arrangements were extracted and a nonlinear minimization [GRG minimizer (Lasdon et al. 1974)] was run on them to obtain a minimum. Then, variations in Al content or the addition of excess enstatite were examined using nonlinear minimization algorithms from the established starting point. Manual examination of various points was also performed to check if more relaxed points could be found, which they could not. This procedure is not guaranteed to find the thermodynamic minimum, but should find how varying Al or enstatite content should change defect concentrations from a fixed starting point.

### Diffusion rates

To convert concentrations into diffusivities, the method outlined in Muir et al. (Submitted) was used. Mg self-diffusion rates were calculated by Eq. 7:

7 $$D_{{{\text{Mg}}}}^{{{\text{sd}}}} = D_{{{\text{Mg}}}}^{{{\text{Vac}}}} N_{{{\text{Vac}}}} + D_{{{\text{Mg}}}}^{{{\text{Int}}}} N_{{{\text{Int}}}},$$

where ${D}_{\mathrm{Mg}}^{\mathrm{Vac}}$ is the diffusion coefficient of Mg vacancies, ${N}_{\mathrm{Vac}}$ is the concentration of Mg vacancies and the same terms apply for interstitials**.** Diffusion coefficients for Mg vacancies and interstitials were calculated in Muir et al. (Submitted) using a kinetic Monte Carlo algorithm. The concentration of Mg vacancies and interstitials is calculated in this paper using the methods outlined above. In Muir et al. (Submitted), it was shown that Mg vacancy diffusion was highly anisotropic (primarily proceeding in the [001] direction) and that Mg interstitial diffusion was fairly isotropic. The overall anisotropy of Mg diffusion in forsterite depends, therefore, upon the balance of vacancy vs interstitial diffusion and their relative concentrations. In this study, the variation of diffusion rate comes solely from the variation of vacancy and interstitial concentrations with changing enstatite and aluminium content. Therefore, the effect of enstatite and aluminium can be considered a multiplier on the diffusion rates presented in Muir et al. (Submitted).

## Results and discussion

### Point defects in forsterite

To calculate the effect of external components on the vacancy content of forsterite, and thus Mg diffusion, we first need to know the intrinsic sources of defects. These can be represented with a series of reactions (R1–R11) constructed using Kröger–Vink notation (Kroger and Vink 1956). The first nine reactions represent all the intrinsic defect-forming reactions in forsterite. R10 represents the addition of MgSiO_3_, and R11 a potential co-effect of aluminium and enstatite. This is discussed in more detail below. An alternate notation scheme (depicting the actual unit cells used in the calculation) is presented in the supplementary information.

$${\mathrm{R}1)\mathrm{ Mg}}_{\mathrm{Mg}}^{X}\to {V}_{\mathrm{Mg}}^{{^{\prime}}{^{\prime}}}+{\mathrm{Mg}}_{I}^{\bullet \bullet},$$

$${\mathrm{R}2)\mathrm{ O}}_{\mathrm{O}}^{X}\to {V}_{O}^{\bullet \bullet }{+\mathrm{O}}_{I}^{{^{\prime}}{^{\prime}},}$$

$${\mathrm{R}3)\mathrm{ Si}}_{\mathrm{Si}}^{X}\to {V}_{Si}^{{^{\prime}}{^{\prime}}{^{\prime}}{^{\prime}}}+{\mathrm{Si}}_{I}^{\bullet \bullet \bullet \bullet },$$

$${\mathrm{R}4)\mathrm{ Mg}}_{\mathrm{Mg}}^{X}+{\mathrm{O}}_{O}^{X}\to {V}_{\mathrm{Mg}}^{{^{\prime}}{^{\prime}}}{+V}_{O}^{\bullet \bullet }+\mathrm{MgO},$$

$$\mathrm{R}5)\mathrm{ MgO}\to {\mathrm{Mg}}_{I}^{\bullet \bullet }+{\mathrm{O}}_{I}^{{^{\prime}}{^{\prime}}},$$

$${\mathrm{R}6) 4\mathrm{Mg}}_{\mathrm{Mg}}^{X}+{\mathrm{Si}}_{\mathrm{Si}}^{X}+{4\mathrm{O}}_{\mathrm{O}}^{X}\to {{4V}_{\mathrm{Mg}}^{{^{\prime}}{^{\prime}}}+\mathrm{Si}}_{I}^{\bullet \bullet \bullet \bullet }+{V}_{\mathrm{Si}}^{{^{\prime}}{^{\prime}}{^{\prime}}{^{\prime}}}+{4V}_{\mathrm{O}}^{\bullet \bullet }+4\mathrm{MgO},$$

$${\mathrm{R}7)\mathrm{ Si}}_{\mathrm{Si}}^{X}+2{\mathrm{O}}_{\mathrm{O}}^{X}+2\mathrm{MgO}\to {V}_{\mathrm{Si}}^{{^{\prime}}{^{\prime}}{^{\prime}}{^{\prime}}}+2{V}_{\mathrm{O}}^{\bullet \bullet }+{\mathrm{Mg}}_{2}{\mathrm{SiO}}_{4},$$

$${\mathrm{R}8)\mathrm{ Si}}_{\mathrm{Si}}^{X}+4\mathrm{MgO}\to {V}_{\mathrm{Si}}^{{^{\prime}}{^{\prime}}{^{\prime}}{^{\prime}}}+2{\mathrm{Mg}}_{I}^{\bullet \bullet }+{\mathrm{Mg}}_{2}{\mathrm{SiO}}_{4},$$

$${\mathrm{R}9) 2\mathrm{Mg}}_{\mathrm{Mg}}^{X}+{\mathrm{Si}}_{Si}^{X}+{4O}_{O}^{X}\to {2V}_{\mathrm{Mg}}^{{^{\prime}}{^{\prime}}}+{V}_{\mathrm{Si}}^{{^{\prime}}{^{\prime}}{^{\prime}}{^{\prime}}}+4{V}_{O}^{\bullet \bullet }+{\mathrm{Mg}}_{2}{\mathrm{SiO}}_{4},$$

$${\mathrm{R}10)\mathrm{ Mg}}_{\mathrm{Mg}}^{X}+{\mathrm{O}}_{O}^{X}+{\mathrm{MgSiO}}_{3}\to {V}_{\mathrm{Mg}}^{{^{\prime}}{^{\prime}}}+{V}_{O}^{\bullet \bullet }+{\mathrm{Mg}}_{2}{\mathrm{SiO}}_{4},$$

$${\mathrm{R}11) 2\mathrm{Al}}_{\mathrm{Mg}}^{\bullet }+{2\mathrm{Al}}_{\mathrm{Si}}^{{^{\prime}}}+{4\mathrm{Mg}}_{\mathrm{Mg}}^{X}+8{\mathrm{MgSiO}}_{3},\to 4{\mathrm{Al}}_{\mathrm{Mg}}^{\bullet }+2{V}_{\mathrm{Mg}}^{{^{\prime}}{^{\prime}}}+{2\mathrm{Si}}_{\mathrm{Si}}^{X}+6{\mathrm{Mg}}_{2}{\mathrm{SiO}}_{4}.$$

At any given temperature and pressure, the energy of each reaction (Δ*G*_reaction_) is calculated which contains the change in both the enthalpy and the lattice vibration entropy. This reaction scheme assumes that the crystal does not develop a charge during any defect production. The starting position of Al in R11 is discussed below.

The presentation of the reactions in Kröger–Vink notation above uses the traditional formulation where the number of sites remain fixed in the defective crystal. This is an assumption (that the concentration of defects is extremely low) but one that makes it impossible to derive a free energy minimization. The reason for this is discussed in the supplementary information, but it means that in our calculations the excess forsterite created by R7–R11 must be allowed to grow the crystal for thermodynamic consistency and increase the number of Mg, Si and O sites. In practice this does not matter, however, as the number of sites in the base crystal changes by < 1% due to R7–R11. We confirmed that this was a negligible effect by finding the local minimum of the free energy when forsterite sites in the crystal are fixed (by introducing an arbitrary term into our equations to account for forsterite site fixing) and this fixed local minimum was found to be the same as the actual global minimum i.e. the change introduced into our calculations by fixing the number of forsterite sites is smaller than the accuracy of our solver.

The energies of reactions R1–R11 are listed in Tables 1 and S3. In the absence of Al at all pressures and temperatures examined, R1 (which creates an Mg Frenkel defect) is the most favourable reaction. This preference for R1 is relatively large (1–3 eV). Considering each reaction in an isolated system, R1 would be favoured by at least three orders of magnitude over the next most favourable reaction (R4) and by over ten orders of magnitude more than the other reactions. This becomes even more pronounced when free energy minimization is considered, as R1 proceeding forwards prevents the other reactions from proceeding forward due to configurational entropy effects. This is because all of these reactions have large positive energies (which drive the reactions strongly to the left and away from defect production) and only produce defects because of configurational entropy, which increases when the reactions proceed to the right and create defects (increasing the configurational entropy). The equilibrium defect concentration occurs when these two energies balance. If large numbers of defects are already present—as, for example, when considering the case of other reactions in the presence of large amounts of defects created by R1—then the gain in configurational entropy for any reaction proceeding to the right is much lower and so too are the equilibrium concentration of defects. Therefore, all reactions produce fewer defects than they do in isolation, but this is primarily seen through the most favoured defect-producing reaction (R1) suppressing the production of defects of the other, less favoured reactions.

**Table 1:** Energy (in eV) of the most favourable reactions as a function of pressure and temperature without considering configurational entropy

	0 K	1000	1500	2000
R1) Mg Frenkel				
0 GPa	6.43	5.96	5.66	5.37
5	6.54	6.37	6.18	5.94
10	6.70	6.92	6.88	6.75
R4) MgO vacancy				
0	7.47	7.42	7.33	7.23
5	8.18	8.51	8.58	8.60
10	8.83	9.59	9.84	10.00
R10) MgO vacancy with Enst				
0	7.23	7.16	7.05	6.93
5	7.99	8.27	8.32	8.31
10	8.67	9.35	9.58	9.72
R11) Al + enstatite reaction				
0	3.27	3.71	3.74	3.66
5	3.37	4.24	4.52	4.67
10	3.46	4.51	5.07	5.44

Therefore, when considered either in isolation or in a combined thermodynamic system, R2–R3 and R5–R9 can be ignored, as including them changes the Mg vacancy and interstitial concentration by less than 1 × 10^–8^%. Thus, these reactions are omitted in minimizations described in the rest of this paper.

Of particular note is R6, which involves the production of Mg vacancies and Si interstitials. This equation (in a slightly modified form) was invoked to describe the effect of enstatite activity on point defects in forsterite by Stocker and Smyth (1978), and has recently been used to explain the relationship between aSiO_2_ and diffusivity of M-site cations in olivine (Zhukova et al. 2014)**.** In contrast, we find that this reaction is extremely unfavourable, even if rewritten to produce enstatite (which lowers its energy by ~ 1.0–0.6 eV depending upon pressure between 0 and 10 GPa). This is because the production of Si interstitials is extremely unfavourable due to their high charge (4 +). This reaction may be the easiest way to produce Si interstitials but it is still very unfavourable (and so the concentration of Si interstitials should be very low). It is very far from the most favourable way to form Mg vacancies or control enstatite effects. R10 is a considerably more favourable reaction for aSiO_2_ control over the Mg vacancy population.

### The effect of enstatite

The effect of the presence of enstatite (and thus SiO_2_ activity) can be represented with reaction R10. Multiple reactions could be constructed here, but this was chosen as the most energetically favourable reaction between MgSiO_3_ and forsterite defects and all other reactions are reachable via combinations of R10 with R1–R9.

As shown in Table 1, R10 is slightly more favourable than R4 (both of which form Mg and O vacancies), but R1 (Mg Frenkel defect formation) is still far more favourable than either of them. Thus, the addition of enstatite to pure forsterite causes effectively no change in the concentration of Mg vacancies or interstitials. Moreover, reaction R1 proceeding forwards largely suppresses the forward procession of R4 and R10 (due to configurational entropy effects). Therefore, even in the presence of enstatite, Mg vacancies in forsterite are essentially entirely formed by R1. Under all conditions tested, the change to the concentration of Mg vacancies by the addition of enstatite was < 0.00001%. Simply put, the addition of enstatite should not affect Mg vacancy concentrations in forsterite and, as a result, SiO_2_ activity should have no effect on Mg (or other M-site cation) diffusion in forsterite, in contrast to the experimental data (Jollands et al. 2020).

### The effect of aluminium

Natural mantle olivine generally contains between ~ 0.001 and ~ 0.1 wt% Al_2_O_3_ (De Hoog et al. 2010), and Al was the main contaminant (10 s wt. ppm) in the nominally pure synthetic forsterite employed in several recent diffusion studies (Zhukova et al. 2014; Jollands et al. 2020). Aluminium in forsterite is generally considered to exist as Tschermak’s defects (Evans et al. 2008; Grant and Wood 2010) where 2 Al^3+^ cations occupy an Mg^2+^ and a Si^4+^ site, respectively. Though the production of Tschermak’s defects is sensitive to pressure and the presence of other charged species (Zhang and Wright 2010; Berry et al. 2007), we find that the Tschermak’s defect is the most stable Al defect of those tested and thus use it as the starting point for our calculations (left hand side of R11).

There is no obvious reason that Al in the Tschermak’s configuration will affect the formation of Mg defects except through modifying the configurational entropy of defects that exist on Mg sites where Al also exists. This has the effect of lowering the relative gain in configurational entropy caused by forming Mg defects and thus suppresses their formation. This effect, however, was also found to be very small (< 0.0001%) due to the small number of Mg defects that do form and thus the presence of Al, in isolation, does not affect the formation of Mg defects and thus does not affect Mg diffusion.

### The co-effect of aluminium and enstatite on defect concentrations

The presence of aluminium alongside enstatite, however, enables a new reaction which creates Mg vacancies and thus affects Mg diffusion (R11). In this reaction, Al starts in the Tschermak’s defect. One Al moves from a Si site to an Mg site with charge balance accommodated by the formation of Mg vacancies. This reaction consumes Si and ejects Mg from the forsterite and so turns enstatite (or equivalently SiO_2_) into forsterite. A version of this reaction which produces MgO instead of consuming enstatite is possible and can be represented as R11 + (R4–R10) × 8, but is much less favourable—the production of MgO is not favoured under these conditions (see Table S2). Zhukova et al. (2017) also demonstrated the presence of a fast-diffusing defect that was hypothesized to occur via an R11-equivalent reaction (using SiO_2_ rather than MgSiO_3_) that was dependant on SiO_2_ activity.

One potential hurdle is that R11, as written, can only occur at the edge of the crystal where forsterite is in contact with free enstatite, or else must involve exsolution. The latter is unlikely due to the positive enthalpy of R11 when compared to the negative enthalpy of forming forsterite from MgO + MgSiO_3_, but it may be favoured by kinetics. A reaction on the surface would be limited by the diffusion rate of Al and Mg vacancies back into the bulk, as otherwise an excess of defects at the grain boundaries would occur. Such diffusion is likely to be faster than bulk diffusion of Mg, however, due to a high local concentration of vacancies at the surface created by R11 which in turns leads to electrostatic repulsion of vacancies into the bulk.

R11, while being generally unfavourable, is much more favourable than any of the intrinsic defect reactions (by at least 1.4/2.1/2.7 eV at 1500 K at 0/5/10 GPa, Table 1) and thus will strongly affect Mg diffusion. This is accomplished both by creating more Mg vacancies and by reducing Mg interstitials through suppressing R1 (Mg-Frenkel defect formation) through configurational entropy effects as explained above for the pure intrinsic system.

**Fig. 1:**
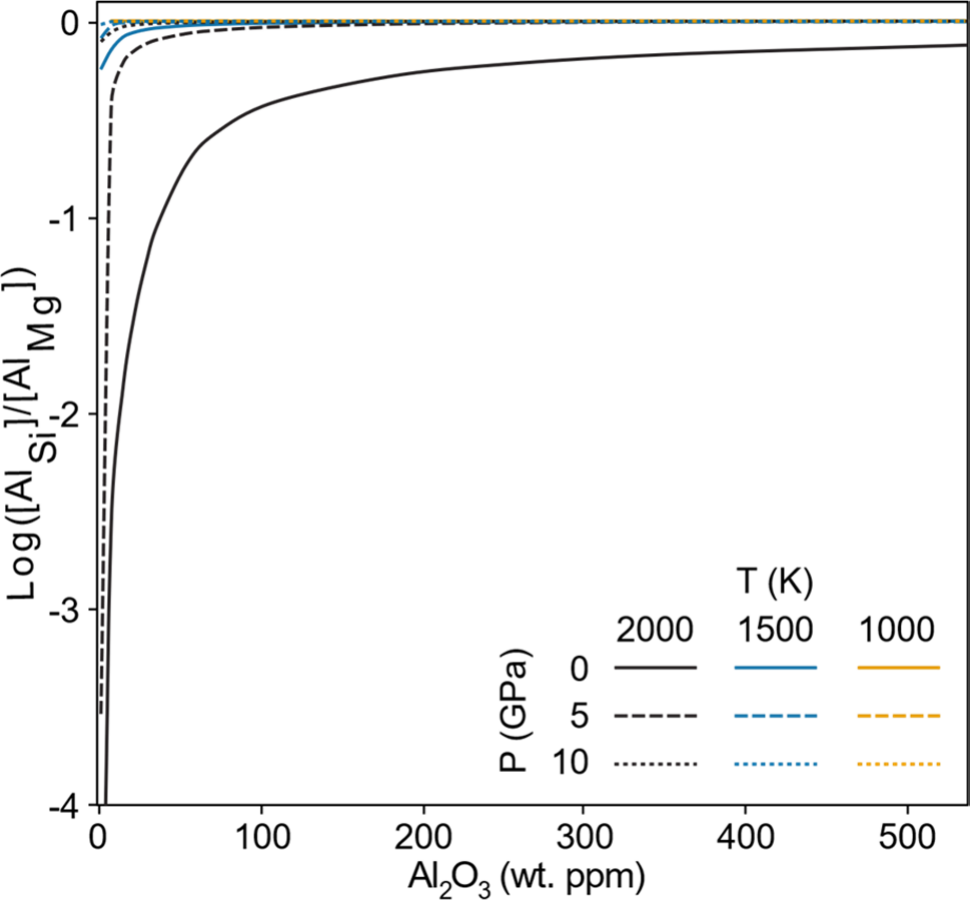
Concentration of Al in silicon sites over concentration of Al in Mg sites as a function of pressure, temperature and aluminium content. Lines are at different pressures and temperatures with shading representing the temperature and solid/dashed/dotted lines representing the pressure. The perfect Tschermak’s defect has a ratio of 0 (log 1). At 1000 K and any pressure or 1500 K and 0 GPa, the line is not distinguishable but is flat at 0

The co-presence of Al and enstatite in association with forsterite leads to two major effects. First, the presence of enstatite changes the distribution of Al between the Mg and Si sites (Fig. 1). As R11 proceeds in the forward direction, it converts ${\mathrm{Al}}_{\mathrm{Si}}^{^{\prime}}$ into ${\mathrm{Al}}_{\mathrm{Mg}}^{\bullet }$ and thus decreases the ${\mathrm{Al}}_{\mathrm{Si}}^{^{\prime}}$:${\mathrm{Al}}_{\mathrm{Mg}}^{\bullet }$ ratio. The progress of this reaction is driven by configurational entropy. This is because converting a Tschermak’s defect (one Mg site defect plus one Si site defect) into three defects on the Mg sites causes a large increase in the number of possible configurations, and thus in configurational entropy. R11 is therefore increasingly favoured by increasing temperature, and so with increasing temperature a higher proportion of Al exists on Mg sites. Increasing the pressure suppresses this reaction as this transition is associated with an increase in volume. This effect is thus only prominent at low pressures and high temperatures (Fig. 1). At high pressures or low temperatures, the overwhelming majority of Al remains in Tschermak’s defects.

**Fig. 2:**
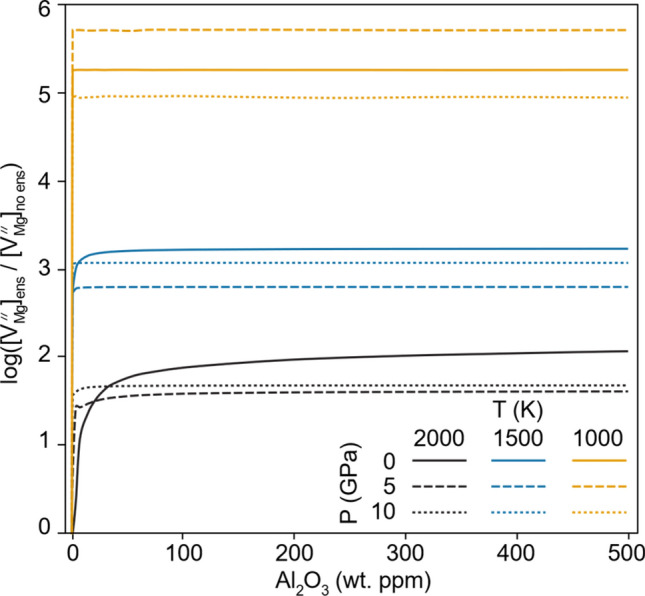
Ratio of Mg vacancy concentrations produced in an aluminous + enstatite system vs those produced in an aluminous only system as a function of Al concentration in wt. ppm Al_2_O_3_. Lines are at different pressures and temperatures with shading representing the temperature and solid/dashed/dotted lines representing the pressure

**Fig. 3:**
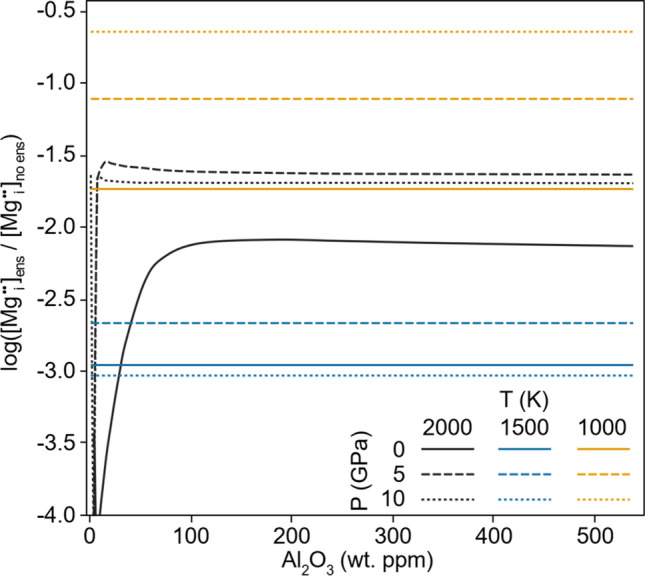
As in Fig. 2 but with the ratio of Mg interstitial concentration instead of Mg vacancy concentration

Second, the combination of Al and enstatite causes a significant increase in Mg vacancy concentration (Fig. 2) and a decrease in concentration of Mg interstitials (Fig. 3). These changes are large, with Mg vacancy concentrations increasing by one to two orders of magnitude (with respect to the enstatite-free system) at 2000 K, and five to six orders of magnitude at 1000 K. Mg interstitial concentrations also decrease by up to three orders of magnitude, and thus this reaction (R11), not Mg Frenkel generation (R1), will define Mg diffusion in cases where enstatite and Al are present. This has important experimental implications as it suggests periclase buffered experiments study the true intrinsic diffusivity of forsterite—controlled by the Mg Frenkel defects—whereas enstatite buffered experiments have additional pathways of diffusion available.

This enstatite + aluminium effect is strongly temperature controlled and only weakly affected by pressure or Al concentration. Decreasing the temperature increases the effect of enstatite + Al on Mg vacancy concentrations (Fig. 2). At an average temperature of 1500 K. this reaction causes the Mg vacancy concentration to increase by ~ 1000 times. Typically, the concentration of Al has no effect beyond an initial saturation point of < 1 wt. ppm Al_2_O_3_. Even at such a low concentration, there are orders of magnitude more Al atoms than the intrinsic defect concentration. With increasing temperature or decreasing pressure, this Al_2_O_3_ saturation point is pushed to greater concentrations.

Some complicated effects are seen—notably the effect of pressure on vacancy and interstitial concentrations and the effect of temperature on interstitial concentrations (Figs. 2, 3, S2-S3). These somewhat strange trends arise due to the interplay between absolute changes in defect concentrations induced by enstatite and aluminium and the base concentration of defects, which change with pressure and temperature in different ways. Therefore, the relative change of defect concentrations (absolute change divided by the base concentration) does not always demonstrate clear trends. Figure S2 and Figure S3 show the absolute changes of vacancy and interstitial concentrations rather than the relative changes shown in Figs. 2, 3. When visualized as an absolute change in defect concentration, pressure clearly increases the effect of enstatite and aluminium on the defect concentrations. Temperature, on the other hand, clearly decreases the effect of enstatite and aluminium. The temperature effect is much larger than the pressure effect. Overall, however, when considering the relative effect of enstatite and aluminium on the defect concentration, decreasing the temperature increases the effect of enstatite and aluminium and changing the pressure has little effect.

### The co-effect of aluminium and enstatite on diffusion and its anisotropy

**Fig. 4:**
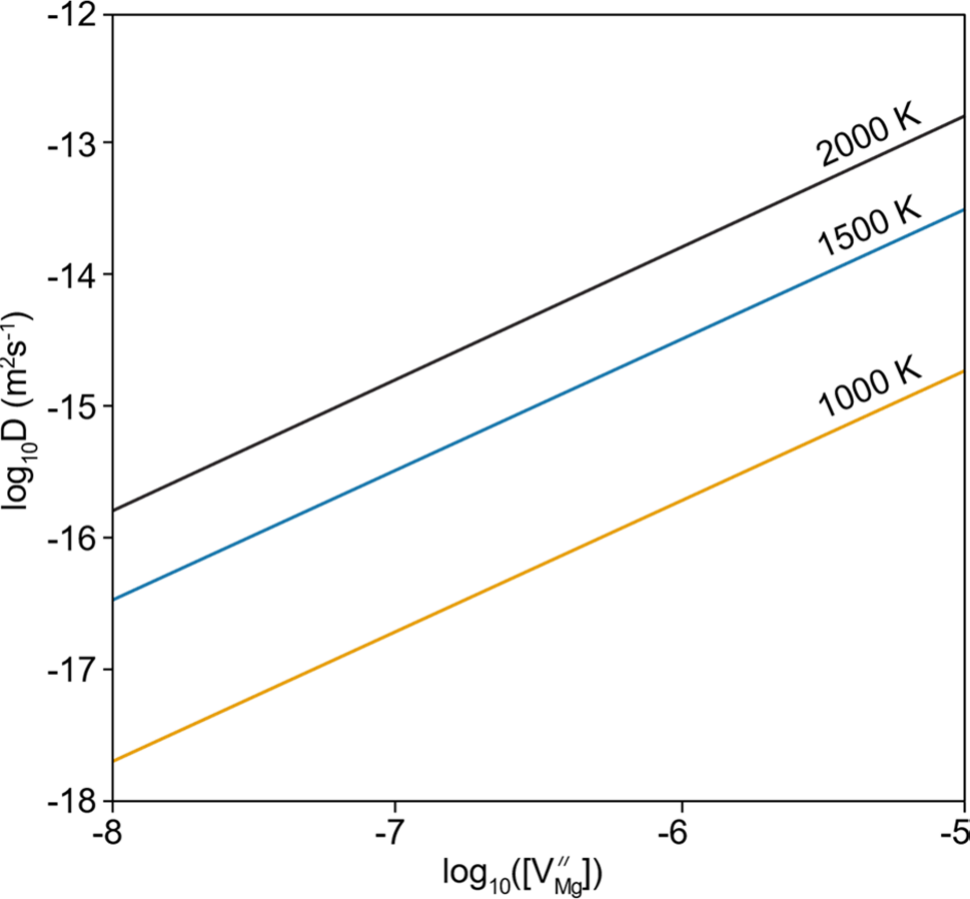
Diffusion rates in periclase-buffered pure forsterite at 0 GPa as a function of vacancy Mg concentration. In each case an equal number of interstitials was added to the system so it was assumed this is a system dominated by R1 (Frenkel defects)

Converting from defect concentrations into diffusion rates is not straightforward, because as shown in Muir et al. (Submitted), both Mg vacancies and Mg interstitials are important for Mg diffusion. Moreover, the relative importance of each of these two components on diffusion depends on pressure and temperature. This is demonstrated in Figure S4 for pure forsterite. Diffusion coefficients for Mg vacancies and interstitials at various pressures and temperatures were determined in Muir et al. (Submitted) by using constrained optimization DFT and these shall be used in this work**.** At each temperature and pressure we take two diffusion coefficients, one each for Mg vacancies and Mg interstitials, from Muir et al. (Submitted), multiply each coefficient by the concentration of that defect at that pressure and temperature to determine the diffusion rate for that defect, and then add together the diffusion rate for each defect to determine the total Mg diffusion rate (Eq. 7). This is demonstrated in Fig. 4 where we show Mg diffusion rates in pure forsterite as a function of defect concentration to stress how fundamental defect concentrations are to overall diffusion rate.

**Fig. 5:**
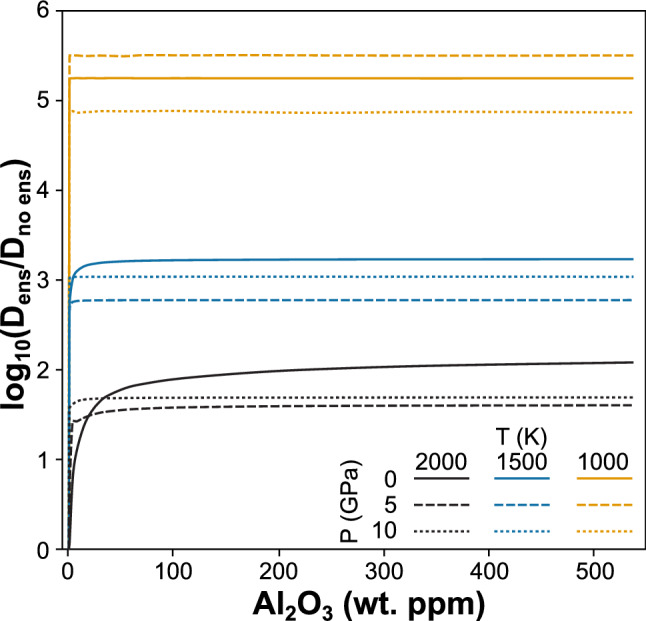
Ratio of absolute diffusion rate between aluminous forsterite with and without enstatite as a function of Al concentration, pressure and temperature. Lines are at different pressures and temperatures with shading representing the temperature and solid/dashed/dotted lines representing the pressure

Figures 5 and S5 show the change in diffusion rates induced by enstatite as a function of Al content. These plots show that the change in diffusion rates caused by enstatite largely track the change in Mg vacancy concentrations caused by enstatite (seen in Fig. 2), while the change in Mg interstitial concentrations caused by enstatite have little effect. This is largely due to the relative changes in the vacancy concentration caused by enstatite and aluminium being much larger than the relative changes in the interstitial concentration. The overall effect, therefore, of enstatite in an aluminous system is a large increase in Mg diffusion rates.

**Fig. 6:**
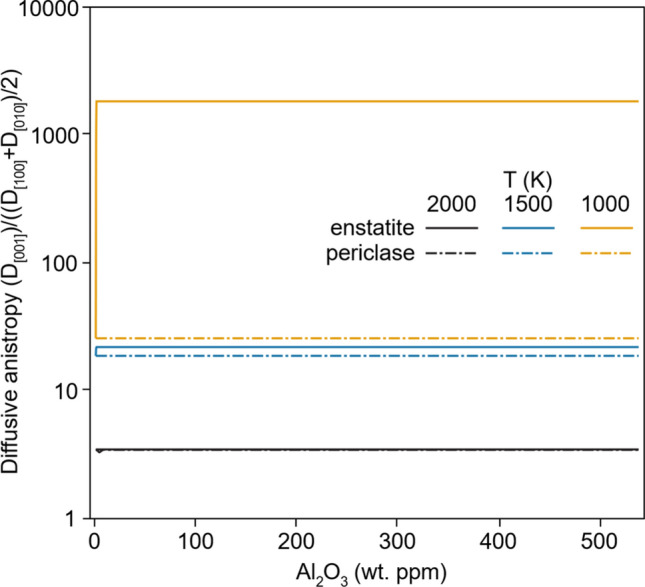
Plot of the preference for [001] diffusion (compared to the average of [010] and [100] diffusion as a function of Al_2_O_3_ content at 0 GPa and different temperatures (solid/dashed/dotted lines). Darker shades represent diffusion buffered by enstatite and lighter shades by periclase. At 2000 K, these two cases are nearly identical, at 1500 K there is little diffusional anistropy difference between enstatite and periclase buffers, at 1000 K this difference is nearly two orders of magnitude

A relative increase in Mg vacancy concentrations (compared to Mg interstitial concentrations) should lead to an increase in the anisotropy of diffusion, with [001] diffusion being favoured (Muir et al. Submitted). This is plotted in Fig. 6 (with additional pressures in Figure S6 and S7). We see that at 2000 K enstatite and aluminium induce no noticeable change in diffusional anisotropy, whereas at 1000 K enstatite and aluminium induce a very strong increase in relative diffusion parallel to [001] (compared to diffusion parallel to [100] and [010]), leading to a strong (nearly 2 orders of magnitude) increase in diffusional anisotropy. This suggests that it may not always be reasonable to apply global fits to Arrhenius relationships using datasets from experiments conducted in different silica activity conditions. In such global fits, the activation energy for diffusion is kept constant and only the pre-exponential factor changes. Whilst such fits may appear reasonable over limited experimental temperature ranges, this has serious implications for the down-temperature extrapolation of experimental diffusion coefficients.

### Site occupancy of Al

In this work, we predict that a major defect forming reaction in forsterite, in the presence of Al_2_O_3_ (> ~ 1 wt. ppm) and enstatite, is a reaction that moves trace Al from Si to Mg sites, and consumes enstatite. This assertion may be tested by examining the coordination number of Al in real forsterite. In recent nuclear magnetic resonance (NMR) experiments (McCarty and Stebbins 2017) a ratio of 1:3 was determined for AlO_4_:AlO_6_ (i.e. tetrahedral to octahedral Al) in samples that had been heated to high temperature (~ 1800 K) with moderate Al contents (320 wt. ppm Al_2_O_3_) and at ambient pressure. Without R11, or equivalent, this ratio should be 1:1 (i.e. representing the Tschermak’s defect) and so a ratio of 1:3 suggests either a reaction that drives the conversion of ${\mathrm{Al}}_{\mathrm{Si}}^{{^{\prime}}}$ into ${\mathrm{Al}}_{\mathrm{Mg}}^{\bullet }$, or that it was never in the ${\mathrm{Al}}_{\mathrm{Si}}^{{^{\prime}}}$ configuration to begin with. These experimental conditions are those in which R11 is significantly activated and we find a similar ratio (1:2), although this ratio would be highly dependent on pressure and temperature. As discussed in Zhukova et al. (2017), any Al incorporation mechanisms leading to a preference of ${\mathrm{Al}}_{\mathrm{Mg}}^{\bullet }$ over ${\mathrm{Al}}_{\mathrm{Si}}^{{^{\prime}}}$ may be more active in real systems than basic thermodynamic equilibration equations suggest, as the production of Tschermak’s defects requires the diffusion of Si, which is slow (Dohmen et al. 2002, Chakraborty 2010). Thus, there is some evidence that an effect similar to R11 exists in real samples. This could be further tested by determining its pressure/temperature/Al content dependence of which the latter is the easiest in NMR.

It should be noted that in the experiments of McCarty and Stebbins (2017), aluminous forsterite was produced from SiO_2_, spinel and MgO, with an MgO excess (1–3 wt%). This MgO excess should suppress, or prohibit, the formation of enstatite. There is an MgO equivalent of R11- (R11 + (R4–R10) × 8—see Table S2), but it has a much weaker effect than R11 and under these conditions produces a 1:1 tetrahedral:octahedral ratio of Al according to our calculations and thus should not display the NMR patterns seen in the experiment. Interpreting the exact reactions that are occurring in this experiment is not straightforward, however, and there are possible ways that Al in forsterite could be exposed to SiO_2_-containing compounds. Firstly, no periclase was detected at the end of the experiment and instead a silicate glass was formed. This suggests that the MgO was possibly not in excess and thus either some enstatite was available to catalyse R11 or that the glass phase had an SiO_2_ excess which could catalyse R11. Secondly, MgO, SiO_2_ and spinel were all ground together so that when spinel dissolves into forsterite, SiO_2_ is likely present. Therefore, these reactions could potentially depend on the kinetics of forsterite formation from SiO_2_ and MgO vs spinel dissolution into forsterite, followed by R11. Further, there is no clear mechanism where the dissolution of spinel would lead to an excess of Al on the Mg sites in forsterite without some sort of SiO_2_ analogue reaction, so it seems likely that some enstatite was present to catalyse the R11 reaction (or something similar).

### Comparison of diffusion rates with literature

**Fig. 7:**
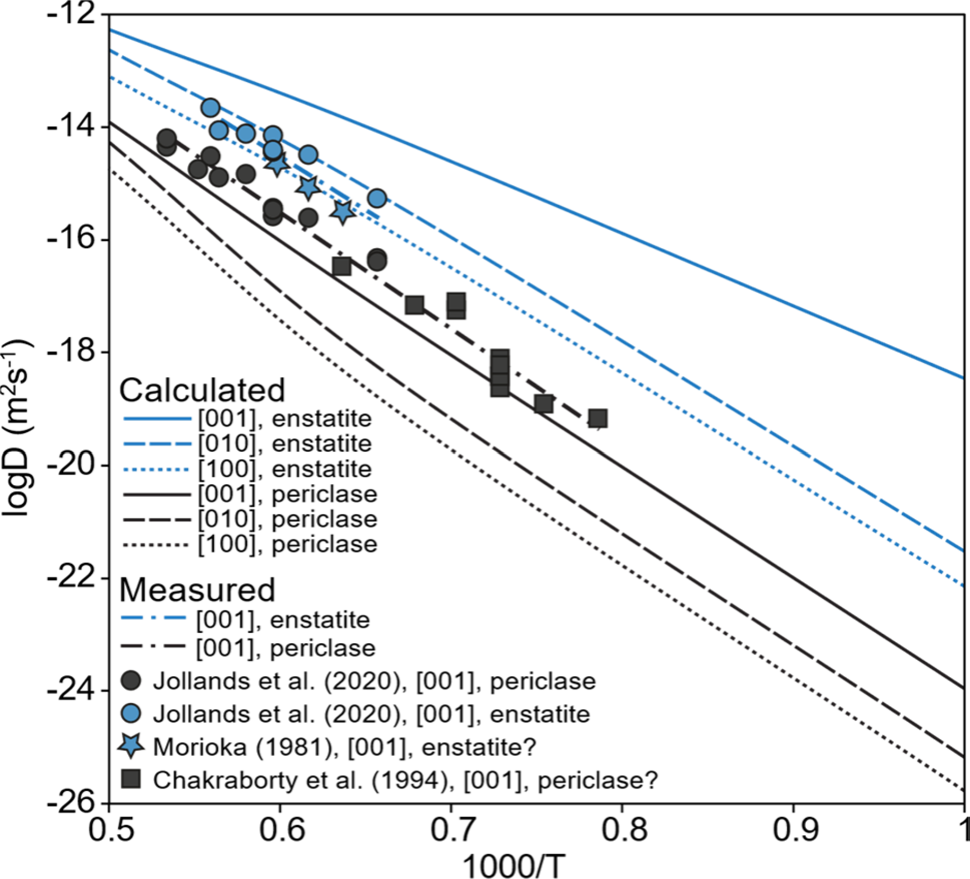
Comparison of our diffusion rates (at 0 GPa) (solid, dotted and dashed lines representing different directions, shade representing buffer chemistry) with experimentally determined diffusion rates (symbols and dotted + dashed line)

In Fig. 7, the calculated diffusion rates are compared with some experimental data. There is a major problem with comparing calculated diffusion rates with experimental diffusion rates, which is that real crystals will have some quantity of extrinsic (impurity-associated) defects that are not accounted for in our model. Furthermore, the type and quantity of these extrinsic defects will vary between different crystals and conditions. Our calculated rate is a baseline from which extrinsic vacancies will causes diffusivities measured in real crystals to deviate. Typically, this will be associated with an increased number of Mg vacancies and thus an increased diffusion rate. However, suppression of the number Mg vacancies (and thus decreasing of the diffusion rate) is also possible. The fact that our results are fairly close to experimental results—and that experimental results are close to each other—suggests that extrinsic vacancies do not play a significant role in experimental diffusion either because they are low in concentration or because they are not involved in the diffusion mechanism with the latter explanation being more likely.

Following the arguments in Jollands et al. (2020), we assume that the Chakraborty et al. (1994) no-buffer data is equivalent to periclase-buffered experiments (MgO film source) and the Morioka (1981) data were enstatite buffered (quartz crucibles). In the periclase-buffered case, we find that our diffusion rate is slightly slower than that seen experimentally. This case is also examined in Muir et al. (Submitted)**.** This is unsurprising as our prediction of diffusion in the periclase-buffered case is likely the minimum possible diffusion rate as explained above with the real crystals possessing some quantity of extrinsic defects. Conversely, our predictions of enstatite-buffered diffusion are somewhat faster than that seen experimentally and this discrepancy has the same cause. Diffusion in the enstatite-buffered case can be considered as a base diffusivity (diffusivity in periclase-buffered conditions) plus an addition to this diffusivity, which is caused by the additional Mg vacancies which are added as a result of the presence of enstatite and aluminium. The size of this additional enstatite + aluminium effect is also dependent on the presence of extrinsic vacancies. We shall consider this in more detail later, but our calculated aluminium + enstatite effect in the pure crystal is close to its maximum size. The presence of extrinsic Mg vacancies will decrease the size of the aluminium + enstatite effect. Thus, the presence of a small amount of extrinsic Mg vacancies would cause the diffusion rate to likely increase in the periclase-buffered case and decrease in the enstatite-buffered case which is exactly what is observed in experiments. Small pressure variations caused by the mismatch of DFT and experimental pressures would also have a significant effect—this is due to the large effect of pressure on the Frenkel reaction R1 (Table 1).

As a final point, it should be noted that our agreement with the literature is not necessarily proof of our model’s accuracy. In our model, we assume a perfect equilibrium for point defect concentration, whereas experimental results will always be in some way kinetically limited. This leaves open the possibility of some significant difference in our predictions and experimental measures which are obscured by kinetic barriers. All three of the studies in Fig. 7 (Morioka 1981; Chakraborty et al. 1994; Jollands et al. 2020) use synthetically grown olivine, which is likely to be at thermodynamic equilibrium in at least the conditions it was grown and thus close to equilibrium in the slightly different experimental conditions. A natural measure of this effect would be to compare the results in synthetic forsterite with natural crystals (which will have different starting defect concentrations), but this cannot be easily done with Mg diffusion in forsterite as the natural crystals have Fe which significantly changes the diffusion rates (Chakraborty et al. 1994). We assume that the experimental measures are close to equilibrium due to no strong evidence of experimental time adjusting experimental results, but possess no systematic proof of such.

Our work shows a clear effect of enstatite on Mg diffusion rates in forsterite. This does not necessitate, however, a change in how we generally consider Mg, or Fe–Mg, diffusion rates in natural mantle olivine. Olivine in the upper mantle is buffered by orthopyroxene (high SiO_2_ activity) and most Mg–Fe interdiffusion work (Dohmen et al. 2007; Chakraborty 1997) and Mg tracer diffusion (Fei et al. 2018; Chakraborty et al. 1994) experiments have been performed in conditions of SiO_2_ excess—i.e. similar conditions to the mantle. Instead we point out that it is important to consider chemical environment in diffusion experiments and that changes to these conditions can be critical.

### The effect of other elements

We have shown that the presence of enstatite, which can be generalized to an increase in aSiO_2_, in the presence of aluminium causes a large increase in anisotropic Mg diffusivity in forsterite that is highly temperature dependent. Thus, to properly model Mg diffusivity in mantle olivine, the background chemical environment needs to be considered. Mantle olivine is not pure forsterite and so it is important to consider how the presence of other elements will affect these results. It is impossible currently to consider the thermodynamic properties of every major, minor and trace element, but we can speculate on how various classes of elements will affect the thermodynamic reactions here. The incorporation of most trace elements will not directly create or destroy vacancies or interstitials in forsterite, but will instead change the configurational entropy of the crystal. As outlined above, defect production is the result of interplay between enthalpic and configurational effects. Increasing the background defect concentration changes the latter term, leading to lower intrinsic defect concentrations, and likely does not change the former term as to do so would require the new elements to interact electronically with the defects, which is unlikely. If we purely consider these changes to this latter term, we can model the effects of introducing other elements, without needing to know their electronic effects on the system. It is particularly important to consider the configurational effect of other elements in the system when modelling the aluminium–enstatite reaction as it is mostly driven by configurational entropy differences. Thus, changing the configurational entropy balance of the starting crystal may significantly impair or enhance the reaction.

Therefore, we can consider these other defects in general groups, based on which sites they occupy and thus how they affect the configurational entropy, without considering their electronic effects. The following three generic categories of external defects will be considered: (1) cations that can exist on Mg and Si sites simultaneously (e.g. Fe^3+^); (2) divalent cations that can substitute for Mg^2+^ (e.g. Fe^2+^); and (3) cations that produce additional Mg vacancies (e.g. H^+^). Trivalent cations that exist on Mg and Si simultaneously are unique in that they can also undergo an equivalent reaction to R11. For this first group of elements, we will therefore also consider the electronics of the species in this group that is most likely to be present in olivine-ferric iron.

### Ferric iron

Iron in olivine is predominantly ferrous, but can be converted between ferrous and ferric states by, for example:

$${\mathrm{R}12) 2\mathrm{Fe}}_{\mathrm{Mg}}^{\bullet }+{2\mathrm{Fe}}_{\mathrm{Si}}^{{^{\prime}}}+{4\mathrm{Mg}}_{\mathrm{Mg}}^{X}+8{\mathrm{MgSiO}}_{3}\to 4{\mathrm{Fe}}_{\mathrm{Mg}}^{\bullet }+2{V}_{\mathrm{Mg}}^{{^{\prime}}{^{\prime}}}+{2\mathrm{Si}}_{\mathrm{Si}}^{X}+6{\mathrm{Mg}}_{2}{\mathrm{SiO}}_{4},$$

which is equivalent to R11. This reaction involves ferric iron and is dependent on MgSiO_3_ (or SiO_2_) activity. Another potential reaction is:

$${\mathrm{R}13)\mathrm{ Fe}}_{\mathrm{Mg}}^{X}+{\mathrm{Si}}_{\mathrm{Si}}^{X}+\frac{1}{2}{\mathrm{Fe}}_{2}{\mathrm{SiO}}_{4}+\frac{1}{2}{\mathrm{O}}_{2}\to {\mathrm{Fe}}_{\mathrm{Mg}}^{\bullet }+{\mathrm{Fe}}_{\mathrm{Si}}^{{^{\prime}}}+\frac{3}{2}{\mathrm{SiO}}_{2,}$$

which is dependent upon oxygen fugacity. R12 is even more favourable than R11 (see Table S2 where the energies of this reaction are presented). In the presence of Fe^3+^ and enstatite (or Fe^2+^, enstatite and a high oxygen fugacity), therefore, very similar (though slightly more exaggerated) results as for Al and enstatite should be seen, with similar trends with concentration, pressure and temperature. The presence of large amounts of Fe^3+^ will push R12 to the right, which will in turn push R11 to the left (as they both compete in configurational entropy terms and R12 is more favourable energetically). They both create the same number of Mg vacancies, however, and so the effect on diffusion will be the same.

Modelling this effect is difficult as it is dependent on the Fe^3+^ concentration and its site distribution, which is itself dependent on multiple variables (total Fe concentration, oxygen fugacity, silica activity and temperature). The relationship between the Fe^3+^:Fe^2+^ ratio and these variables is poorly constrained, but, according to the model of Dohmen and Chakraborty (2007) the Fe^3+^ concentration of an olivine crystal with a typical Mg/(Mg + Fe) value of 0.9 (De Hoog et al. 2010) would be around ~ 0.5–500 atomic ppm. Only around 1–10 atomic ppm Fe^3+^ is needed to activate the effect of enstatite on Mg (or Fe–Mg) diffusion and so there is likely enough Fe^3+^ in natural mantle olivine to activate R12. However, a better definition of the Fe^3+^:Fe^2+^ ratio is needed to be certain.

At this point, we would like to discuss another reaction:

$${\mathrm{R}14) 6\mathrm{Fe}}_{\mathrm{Mg}}^{X}+{\mathrm{MgSiO}}_{3}+{\mathrm{O}}_{2}\to 4{\mathrm{Fe}}_{\mathrm{Mg}}^{\bullet }+2{V}_{\mathrm{Mg}}^{{{\prime}}{{\prime}}}+{\mathrm{Fe}}_{2}{\mathrm{SiO}}_{4}+\mathrm{MgO}.$$

R14 has been invoked to provide the majority of defects in natural olivine above 1100 K until an even higher point when pure intrinsic defects take over (Chakraborty 2010) and has been labelled the Transition Metal Extrinsic Diffusion (TaMED) regime**.** It should be noted that the TaMED reaction is typically written with SiO_2_ instead of MgSiO_3_ on the left, but these regimes should be equivalent.

While the exact chemical mechanism by which R14 occurs is unknown, R14 can be carried out in a two-step mechanism by first oxidizing ferrous iron and pushing some of it onto the Si sites (R13) and then eliminating this tetrahedrally coordinated ferric iron through an enstatite reaction (R12). It is highly unlikely that either ferric iron forms entirely on the Mg site or that Mg vacancies form spontaneously in the first step, as these will create unbalanced charges with large energies. The activation barrier of R12 is likely not to be particularly high and R13 has been used in previous point defect models as one of the pathways for the oxidation of ferrous iron in forsterite (Dohmen and Chakraborty 2007) and thus likely also has a low barrier. Therefore, R13 followed by R12 is a plausible two-step mechanism for carrying out R14. We cannot, however, rule out a single-step mechanism by which some iron oxidizes and some iron is expelled to create Mg vacancies in a single step.

If R14 indeed operates by the two-step mechanism proposed above, then our conclusions about the dynamics of R11 and R12 should also hold for R14 and the TAMED mechanism (Chakraborty 2010). **I**n this process, R12 will be an important control on R14. Then, as R12 is more favourable than R11, the overall system should operate in the way that the TAMED regime predicts, when iron, aluminium, enstatite and oxygen are all present. This means that the system's response to changes in external chemistry (such as changes in silica activity and oxygen fugacity) will be controlled by how ferric iron responds to these changes rather than by how aluminium responds, in real cases where both ferric iron and aluminium are present.

### Ferrous iron

We next consider the effect of elements substituting isovalently for Mg^2+^, specifically Fe^2+^. Fe^2+^ was introduced as an inert substitutional defect that does not interact with any of the defect-forming reactions, but instead just changes the configurational entropy balances. Any atom that replaces Mg^2+^ will have a similar effect. The effect of introducing substitutional Mg defects is shown in Figure S8 and S9. Here, we see that this can have a significant impact on the enstatite + aluminium effect (− 15% to 10%) but that this is small relative to the orders of magnitude change that enstatite + aluminium normally induces. Thus, the main conclusion that enstatite and aluminium induce a very large change in Mg diffusion in forsterite is robust, even with large amounts of Fe^2+^ in the system.

### Hydrous vacancies

Finally, we consider the effect of extrinsic Mg vacancies. One of the most plausible sources of Mg vacancies is associated with hydroxyl/hydrogen/’water’. While there is some debate about where exactly hydroxyl resides in olivine, and under what conditions (Matveev et al. 2001; Le Losq et al. 2019; Berry et al. 2005; Tollan et al. 2018, 2017; Lemaire et al. 2004; Mosenfelder et al. 2006, 2011; Padron-Navarta et al. 2014), hydrated Mg vacancies [(2H)_Mg_^X^] are consistently observed by FTIR as low, broad bands in the 3150–3250 cm^−1^ wavenumber region. The exact ratio of water fugacity to hydrous Mg vacancies is difficult to establish. For example, much of the incorporated hydrogen is likely to be associated with octahedral Ti^4+^ and a tetrahedral vacancy ${\mathrm{Ti}}_{\mathrm{Mg}}^{\bullet \bullet }(2\mathrm{H}{)}_{\mathrm{Si}}^{{{\prime}}{{\prime}}}$ (Berry et al. 2007; Walker et al. 2007). However, the exact definition of this ratio is unimportant to this work—more important is the amount of vacancies that are created in the crystal. The amount of $(2\mathrm{H}{)}_{\mathrm{Mg}}^{X}$ vacancies that are created in the system should be a function of the fugacity of water and silica activity and thus can be represented with the following equations:

8 $${\text{Mg}}_{{{\text{Mg}}}}^{X} + {\text{H}}_{2} {\text{O}} + \frac{1}{2}{\text{SiO}}_{2} = (2{\text{H}})_{{{\text{Mg}}}}^{X} + \frac{1}{2}{\text{Mg}}_{2} {\text{SiO}}_{4},$$

9 $$f_{{{\text{H}}_{2} {\text{O}}}} a_{{{\text{SiO}}_{2} }}^{1/2} \propto \gamma [\left( {2{\text{H}})_{{{\text{Mg}}}}^{X} } \right],$$

where $\gamma$ is some value that is roughly constant and represents the effect of other water-forming defects. In this work, we have set this to 1 which assumes no other water-forming defects are important so that only variations in water fugacity are important. This would be the case in a pure forsterite crystal which has no alternative water sinks and with high silica activity so that no $(4\mathrm{H}{)}_{\mathrm{Si}}^{X}$ forms (Walker et al. 2007).

Table S3 shows how adding additional inert vacancies changes the magnitude of the aluminium + enstatite effect. Creating a large number of additional Mg vacancies has a large suppressive effect on the aluminium + enstatite reaction. This is because R11 is driven by configurational entropy gains associated with creating a large number of Mg vacancies, but these gains are much smaller when a large number of Mg vacancies are already present. In this case, the strong positive enthalpy terms of R11 become more important. At 1000 K, even the addition of 0.2 H/Si ppm hydrated Mg vacancies (~ 0.013 wt. ppm H_2_O using Eq. 9 and $\gamma =1$) causes enstatite and aluminium to have no noticeable effect on Mg vacancy concentrations. In contrast, at 2000 K, 1000 H/Si ppm hydrated Mg vacancies (~ 128 wt. ppm H_2_O) are needed to eliminate all effects. This would suggest that at low temperatures, even trace amounts of water can eliminate the enstatite effect, but at high temperatures large amounts of water would be required. In a real crystal, however, this will change because $\gamma$ will be significantly lower than 1. The exact ratio of the concentration where water suppresses this effect on $\gamma$ is complex and has much discussion in the literature (see for example Tollan et al. (2017), Tollan et al. (2018) Walker et al. (2007), Le Losq et al. (2019) and Berry et al. (2005)). While enstatite both enables this reaction and favours $(2\mathrm{H}{)}_{\mathrm{Mg}}^{X}$ over $(4\mathrm{H}{)}_{\mathrm{Si}}^{X}$ formation (Walker et al. 2007), global equilibrium experiments (Tollan et al. 2017) show that $(2\mathrm{H}{)}_{\mathrm{Mg}}^{X}$ is likely a minor product regardless of *P*, *T* and buffer. The maximum amount of water detected at this site is ~ 4 wt. ppm in this experiment (although this is dependent on the absorption coefficient) even when forsterite is buffered by enstatite as other water-bearing defect sites dominate. Only in the absence of contaminants and in the presence of enstatite should $(2\mathrm{H}{)}_{\mathrm{Mg}}^{X}$ be the dominant defect (Walker et al. 2007). Thus in real crystals, $\gamma$ is considerably below 1. This means that the amount of$(2\mathrm{H}{)}_{\mathrm{Mg}}^{X}$, that is formed should be much less than the values required to significantly affect the aluminium + enstatite (R11) reaction. Therefore, the aluminium + enstatite (R11) reaction such not be suppressed by water in real crystals even at low temperatures, as the concentration of $(2\mathrm{H}{)}_{\mathrm{Mg}}^{X}$ will be too low.

## Conclusions

Recent experimental results have shown that the chemical environment of forsterite—in particular aSiO_2_—can affect its diffusional characteristics. In this work we examine the mechanism of this change and how aSiO_2_ specifically affects diffusion through its effect on defect concentrations. The sole addition of enstatite to pure forsterite makes no noticeable difference to the concentration of Mg vacancies and interstitials in forsterite and thus does not perceptibly change its diffusion rates. When forsterite includes even trace amounts of aluminium, or other 3 + cations in a Tschermak’s defect (e.g. ferric iron), these cations can react with enstatite to produce a large increase in Mg vacancy concentration and thus Mg diffusion, primarily favouring diffusion in the [001] direction. This increase is inversely proportional to temperature and not reliant on pressure. This increase can be very large (~ 6 orders of magnitude) in pure forsterite, but is dependent on background chemistry and will be suppressed by significant numbers of external Mg vacancies. Ultimately, this study shows how the chemical environment of forsterite can have a large effect on its Mg-diffusivity and that changes in aSiO_2_ can have profound effects on both the speed and anisotropy of Mg diffusion in forsterite. This means that when applying experimentally determined diffusivities, one needs to consider the chemical environment in which those diffusivities were obtained and whether it matches that of the geological system in question.

## Electronic supplementary material

Below is the link to the electronic supplementary material.

Supplementary file1 (DOCX 1646 KB)
