# Supplementary material for: Explaining the dependence of M-site diffusion in forsterite on silica activity: a density functional theory approach
Source: Phys Chem Miner. 2020 Nov 24;47(12):55. doi: 10.1007/s00269-020-01123-5 (PMC7686175; doi:10.1007/s00269-020-01123-5)
Supplement: Supplementary file 1 — Supplementary file1 (DOCX 1646 KB) [file 269_2020_1123_MOESM1_ESM.docx]

Supplementary Information for Explaining the dependence of M-site diffusion in forsterite on silica activity: a Density Functional Theory approach

In this document we provide some additional data for clarification of the main text. We provide a full breakdown of our configurational entropy calculations and some discussions of its limitations. We provide the actual unit cells used in the calculations of free energy rather than the Kroger-Vink notation that is provided in the main text and we provide some additional text upon the implications of the site balance criterion that is enforced in the standard Kroger Vink notation scheme.

We also provide some alternative plots of our data to ease interpretation- a plot of the absolute (rather than the relative) changes of defects caused by aluminium and enstatite in forsterite (Figure S1 and S2), a plot demonstrating the importance of interstitial diffusion (Figure S3) , a plot comparing the changes in diffusion rate and Mg vacancy concentration caused by aluminium and enstatite (Figure S4), plots of the change in Mg diffusional anisotropy induced by alumium and enstatite at different pressures (Figure S5 and S6| and of the effect of ferrous iron on these defect concentrations (Figure S7 and S8).

We also provide tabulations of the enthalpic preference for defects to sit at different sites in forsterite (Table S4), the pairing energies of some defect charge pairs (Table S1), the energies of less favoured defect reactions (Table S5), the energy of ferric iron equivalents of our aluminium reactions (Table S2) and the change caused to these results by hydrous Mg vacancies (Table S3).

Configurational Entropy Calculations

To solve for the configurational entropy term, we used the Gibbs entropy formula:

$S=-k_{B}\sum_{j} p_{j}lnp_{j}$ Equation S.1

Where k_B_ is the Boltzmann constant, j represents a specific configuration of defects within an arrangement and p_j_ the probability that configuration occurs. The probability of any specific configuration occurring is:

$p_{j}=\frac{1}{Z}e^{(-U_{j}/k_{B}T)}$ Equation S.2

Where U_j_ is the internal energy of each configuration as in Equation 5. Strictly, this should be determined with the free energy (G in Equation 5) of each configuration. However, we have assumed that the largest change in the energy of the different configurations comes from the enthalpy differences between them, thus we have ignored changes to vibrational entropy between different configurations except for Mg interstitials, as outlined below. To approximate the dilute limit we have fixed the volume of all defect cells to that of perfect forsterite. Therefore, the PV term does not change between different configurations, and Equation S.2 is calculated with changes in U only. Z in equation S.2 is the canonical partition function:

$Z=\sum_{i} e^{(-E_{j}/k_{B}T)}$Equation S.3

At this point we shall define a scheme to group the configurations and bring them down to a calculable number. We shall thus define a configuration as a state where each of the defects of each type is confined to a single type of site. This can be imagined by having a single defect of each type and so the different configurations simply change which site each defect occupies. With our assumption that defects are independent every configuration with the defects confined to a single site is identical and configurations where defects occupy multiple sites are included by the partitioning function. With the defects listed above there are 1176 configurations, all of which were tabulated (the full list is not provided as it is elementary and long). Each configuration has many ways that it can be arranged in the cells and this degeneracy is not equivalent for different configurations. To calculate the degeneracy (W) of each configuration, we must first calculate the degeneracy at each site:

W=ln$\frac{N!}{a!b!\ldots z!}$Equation S.3

Where N is the total number of sites, and a,b,c…z are the different types of atoms/defects at each site including a final z term, which is simply (N-a-b….-y). To solve this numerically, all defect concentrations were written in terms of defects/mol and then the Stirling approximation was used ($lnn!\cong nln-n$), giving:

$W=NlnN-N-alna+a-blnb+b\ldots-zlnz+z$ Equation S.4

To calculate the energy (U_j_) of each configuration, the difference in enthalpy between placing a defect at the different sites was calculated as a function of pressure (Table S4), and energy penalties were assigned for defects that were not in their favoured site. As such, we assume in this formulation that the relative energy of placing defects in different sites is not a function of temperature, and temperature effects occur only in differences between different types of defect (such as $V_{Mg}^{''}$ and ${Mg}_{I}^{\cdot\cdot})$and not between the same defects at different sites ($V_{Mg}^{''}$ at M1 and M2 for example) as the different Mg sites are similar and thus their vibrational frequencies should be similar. This was tested for two of the most prominent defects- Mg vacancies and Al in Mg sites- where it was found that the vibrational entropy difference between the two sites was less than 10 meV/defect due to the large similarities between the two sites. One notable exception is Mg interstitials which have quite different geometries between their two sites (M1 and I2) (Muir et al. (Submitted)) and thus quite different distributions of phonon frequencies. The vibrational entropy difference between these two sites is tabulated in Table S4 and included in the model and thus for Mg interstitials we use G rather than U in Equation S.2.

Knowing the degeneracy and relative energy of all configurations, the entropy was calculated using Equation 7 but summed across i, where i is simply a sum across every configuration (j) appearing a number of times equal to its degeneracy (W).

This is further supported by the fact that temperature has only a weak effect on the pairing energy (Table S1) even when defects are adjacent to each other. If we consider the phonons of a system with two defects (1 and 2) as Phonon_system_=Phonon_base_+∆Phonon_defect1_+∆Phonon_defect2_+ ∆Phonon_defect1-2_ then the final term which represents the coeffect of the two defects must be close to 0 or a large temperature effect would be seen on the pairing energy. Therefore the lack of a strong temperature effects shows that the changes that defects make to the phonon of the crystal are largely independent of each other. These conclusions justify both treating configurational entropy as a series of isolated defects arranged randomly throughout the crystal and treating the enthalpy and vibrational entropy of each individual defect as a separate addition to the energy of the forsterite unit cell.

A major assumption in this formulation is that the entropy of mixing is ideal ie that all configurations with the same types and configurations of defects have the same energy. This assumption is predicated on the very small number of total defects relative to the unit cell and the fact that they energetically don’t like to associate with each other (Table S1). Both of these points suggest that defects exist as isolated points and the local conditions around the defect are always those of bulk forsterite which leads to ideal mixing. We have incorporated the most likely major deviation from ideal mixing- the energy changes when defects change site- but we cannot test this assumption rigorously as to do so would require the simulations of millions of atoms. This is therefore a limitation of our method.

Alternate reaction formulations

R1) ${2Mg}_{32}{Si}_{16}O_{64}\to{Mg}_{31}{Si}_{16}O_{64}+{Mg}_{33}{Si}_{16}O_{64}$

R2) ${2Mg}_{32}{Si}_{16}O_{64}\to{Mg}_{32}{Si}_{16}O_{63}+{Mg}_{32}{Si}_{16}O_{65}$

R3) ${2Mg}_{32}{Si}_{16}O_{64}\to{Mg}_{32}{Si}_{15}O_{64}+{Mg}_{32}{Si}_{17}O_{64}$

R4) ${2Mg}_{32}{Si}_{16}O_{64}\to{Mg}_{31}{Si}_{16}O_{64}+{Mg}_{32}{Si}_{16}O_{63}+MgO$

R5)${2Mg}_{32}{Si}_{16}O_{64}+MgO\to{Mg}_{33}{Si}_{16}O_{64}+{Mg}_{32}{Si}_{16}O_{65}$

R6) ${4Mg}_{32}{Si}_{16}O_{64}\to{2Mg}_{31}{Si}_{16}O_{64}+{Mg}_{32}{Si}_{17}O_{64}+{Mg}_{30}{Si}_{15}O_{60}+4MgO$

R7) ${3Mg}_{32}{Si}_{16}O_{64}+2MgO\to{Mg}_{32}{Si}_{15}O_{64}+2{Mg}_{32}{Si}_{16}O_{63}+{Mg}_{2}SiO_{4}$

R8) ${3Mg}_{32}{Si}_{16}O_{64}+4MgO\to{Mg}_{32}{Si}_{15}O_{64}+2{Mg}_{33}{Si}_{16}O_{64}+{Mg}_{2}SiO_{4}$

R9) ${4Mg}_{32}{Si}_{16}O_{64}\to{2Mg}_{31}{Si}_{16}O_{64}+{Mg}_{32}{Si}_{15}O_{64}+{4Mg}_{32}{Si}_{15}O_{63}+{Mg}_{2}SiO_{4}$

R10) ${2Mg}_{32}{Si}_{16}O_{64}+MgSiO_{3}\to{Mg}_{31}{Si}_{16}O_{64}+{Mg}_{32}{Si}_{16}O_{63}+{Mg}_{2}SiO_{4}$

R11) ${2Mg}_{31}Al{Si}_{16}O_{64}+{2Mg}_{32}{Si}_{15}AlO_{64}+{2Mg}_{32}{Si}_{16}O_{64}+8MgSiO_{3}\to{4Mg}_{31}Al{Si}_{16}O_{64}+{2Mg}_{31}{Si}_{16}AlO_{64}+6{Mg}_{2}SiO_{4}$

An alternate way of writing reactions R1-R11- in this case the actual unit cells that were used in the defect calculations are shown.

Comments upon Kroger-Vink site balance:

Free energy can be represented as such:

$dG=-SdT+VdP+\sum_{i=1}^{n} \mu_{i}dN_{i}$ Equation S1

Where i is the different species (ie Mg vacancy, Mg interstitial, forsterite etc) and $\mu_{i}$ is the chemical potential of that species and N_i_ is the concentration of that species. At a set temperature and pressure varying the concentration of the individual species varies G and a minimum G can be found. Chemical potential is defined, however as:

$\mu_{i}=(\frac{\partial G}{\partial N_{i}})_{T,P,N_{j\neq i}}$Equation S2

With a fixed number of sites in the base forsterite, Equation S2 cannot be solved for every species. This is due to the requirement that to determine chemical potential, the concentration of that species must be varied whilst keeping the concentrations of all other species fixed. With a fixed amount of sites this is impossible to achieve as the concentration of forsterite is tied to that of all other species.

If we consider say R11 in the Kroger-Vink notation moving this reaction to the right increases the amount of forsterite and decreases the concentration of other vacancies which renders the evaluation of the chemical potential of the reactants involved impossible. The actual formulation of equations we used to evaluate free energy is listed above in the alternative reaction formulation section. This considers reactants as supercells with built in defects and in this formulation the sites are not fixed in the forsterite base and thus chemical potential can be determined and free energy minimized. As discussed in the text this is a very small point, however, that makes no change to our results and relaxing site balance is simply done because it is required for thermodynamic consistency.


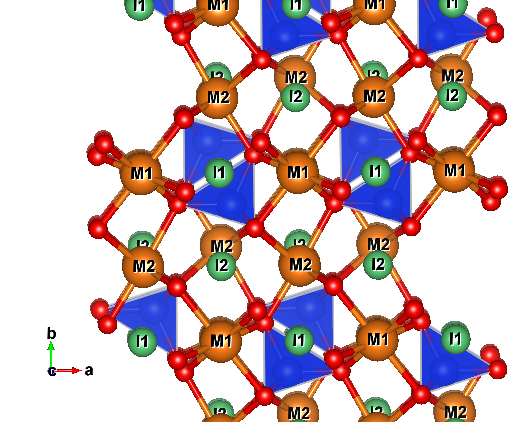


[100]

[010]

Figure S1: Picture of forsterite with certain sites marked. M1 and M2 are Mg occupied octahedral sites, I1 and I2 are unoccupied octahedral holes. Si atoms are marked in blue as is their SiO_4_ tetrahedron with oxygens marked in red. For each Si atom there are 7 tetrahedral holes which can be generated as described in the text.


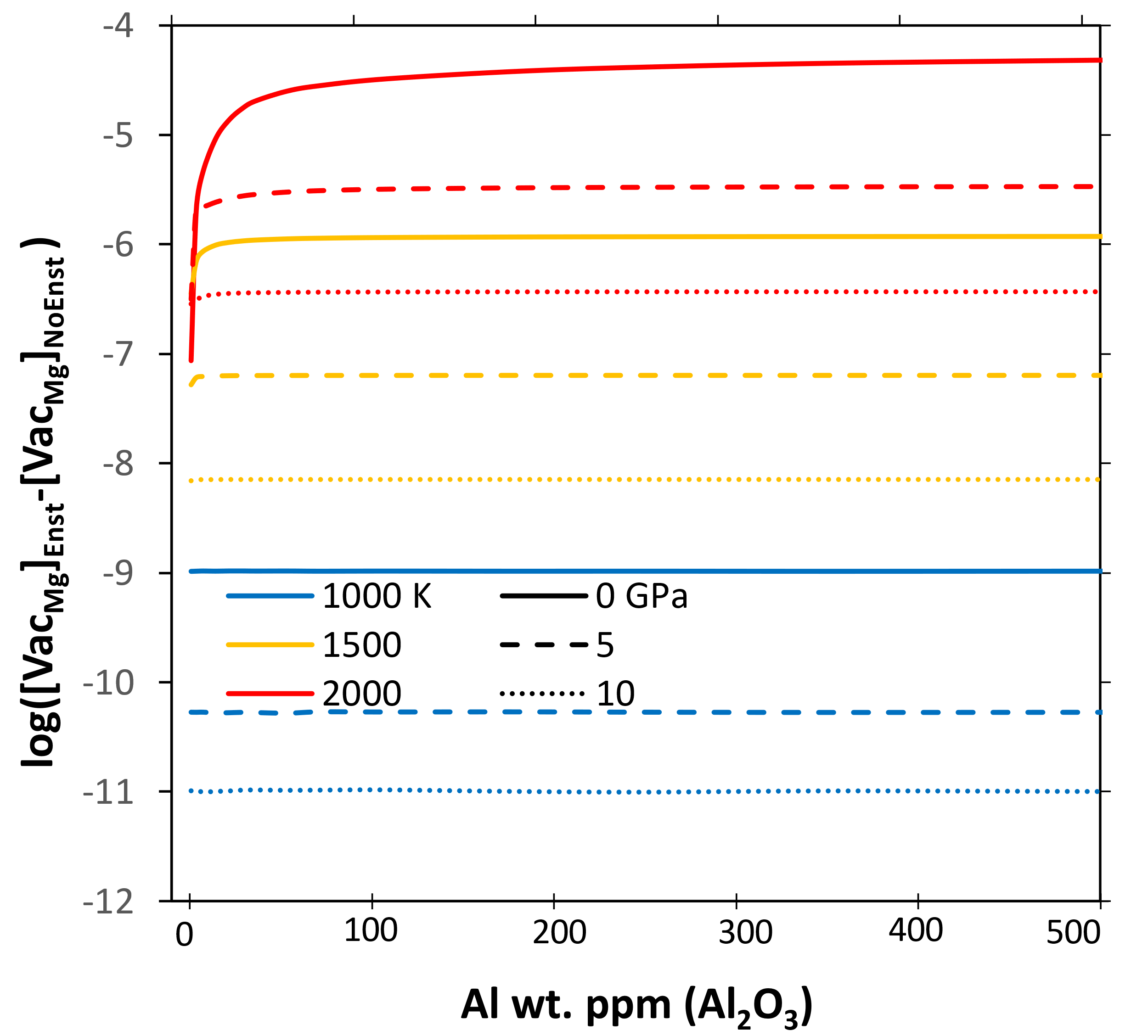


Fig S2- Absolute difference in Mg vacancy concentration between an aluminous+enstatite system vs those produced in an aluminous only system as a function of Al concentration in wt. ppm of Al_2_O_3_. Lines are at different pressures and temperatures with colour representing the temperature and solid/dashed/dotted representing the pressure.


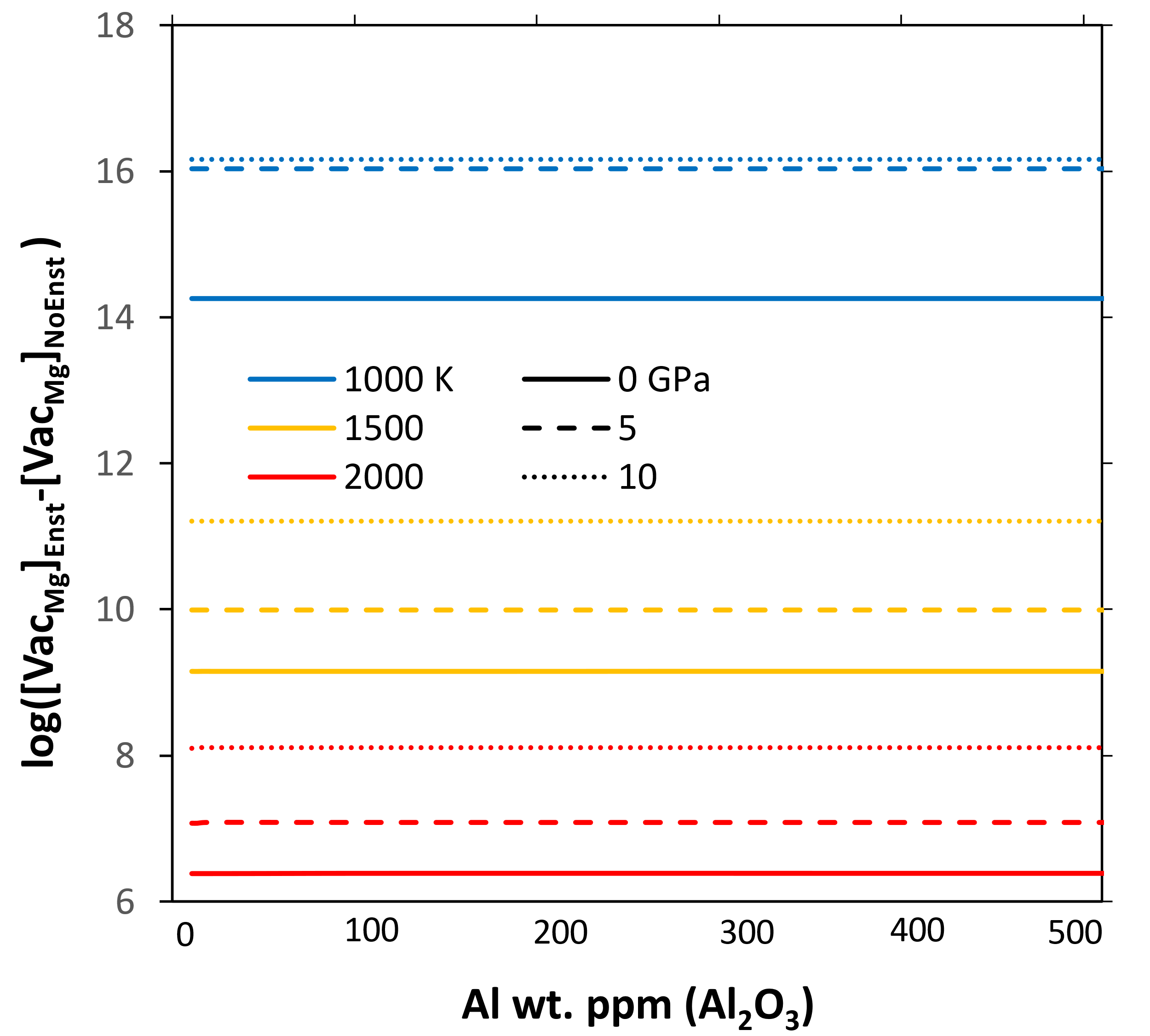


Fig S3- As Figure S2 but for Mg interstitial concentration


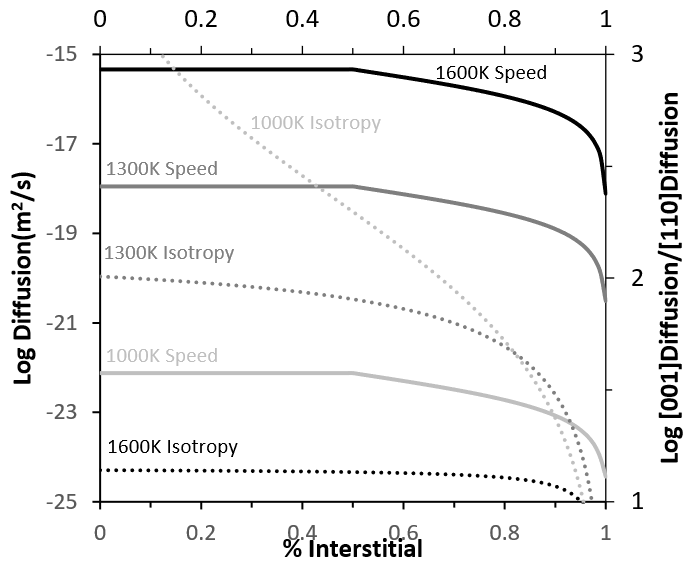


Figure S4: Plot of log of the diffusion rates (left axis, solid lines) and the log of [001] diffusion divided by [110] diffusion as a measure of diffusional anisotropy (right axis, dotted lines) at different temperatures for perfect forsterite as a function of whether interstitial or vacancy diffusion are included in the calculation. This was calculated by first calculating the diffusivity and concentration of Mg vacancies and interstitials. Then we defined $\%Interstitial=\frac{D{Mg}_{I}^{\bullet\bullet}}{(DV_{Mg}^{''}+{DMg}_{I}^{\bullet\bullet})}$ where D is the diffusion of those species. In this way at 0% only vacancy diffusion is switched on, at 100% only interstitial diffusion is switched on and at 50% vacancy and interstitial diffusion are fully switched on (50% represents the real case in pure forsterite). This graph was plotted for data at 0 GPa.

As can be seen interstitials make only small differences to the diffusion rate as vacancy diffusion is slightly faster but make large changes to the diffusion anisotropy. Increasing the number of interstitials decreases the anisotropy of diffusion with this decrease being smaller with increasing temperature.


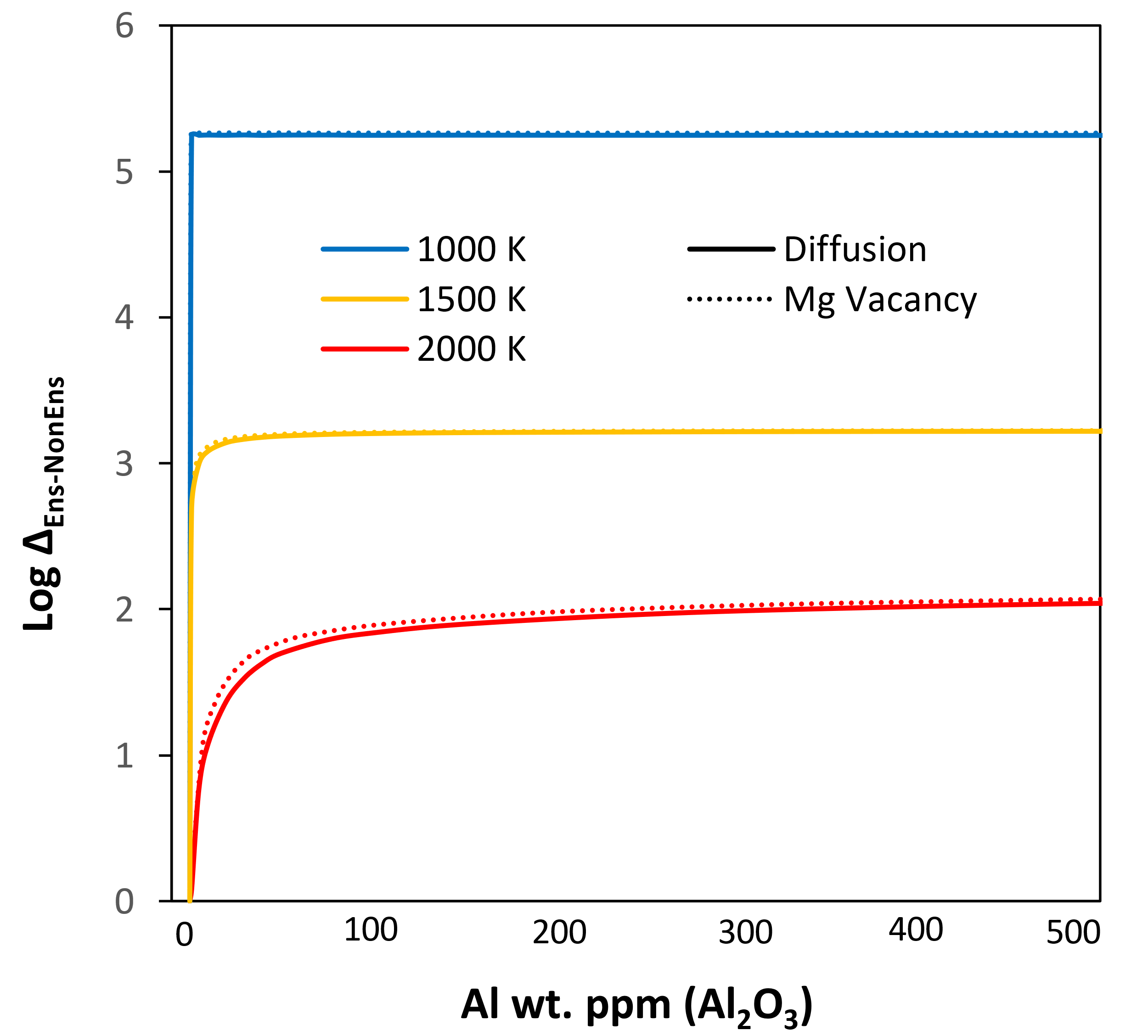


Figure S5- Comparision of the change induced in diffusion rate (in m^2^/s solid line) and Mg vacancy concentration (in defects/unit cell dotted line) by enstatite as a function of temperature (colour) and Al content at 0 GPa.


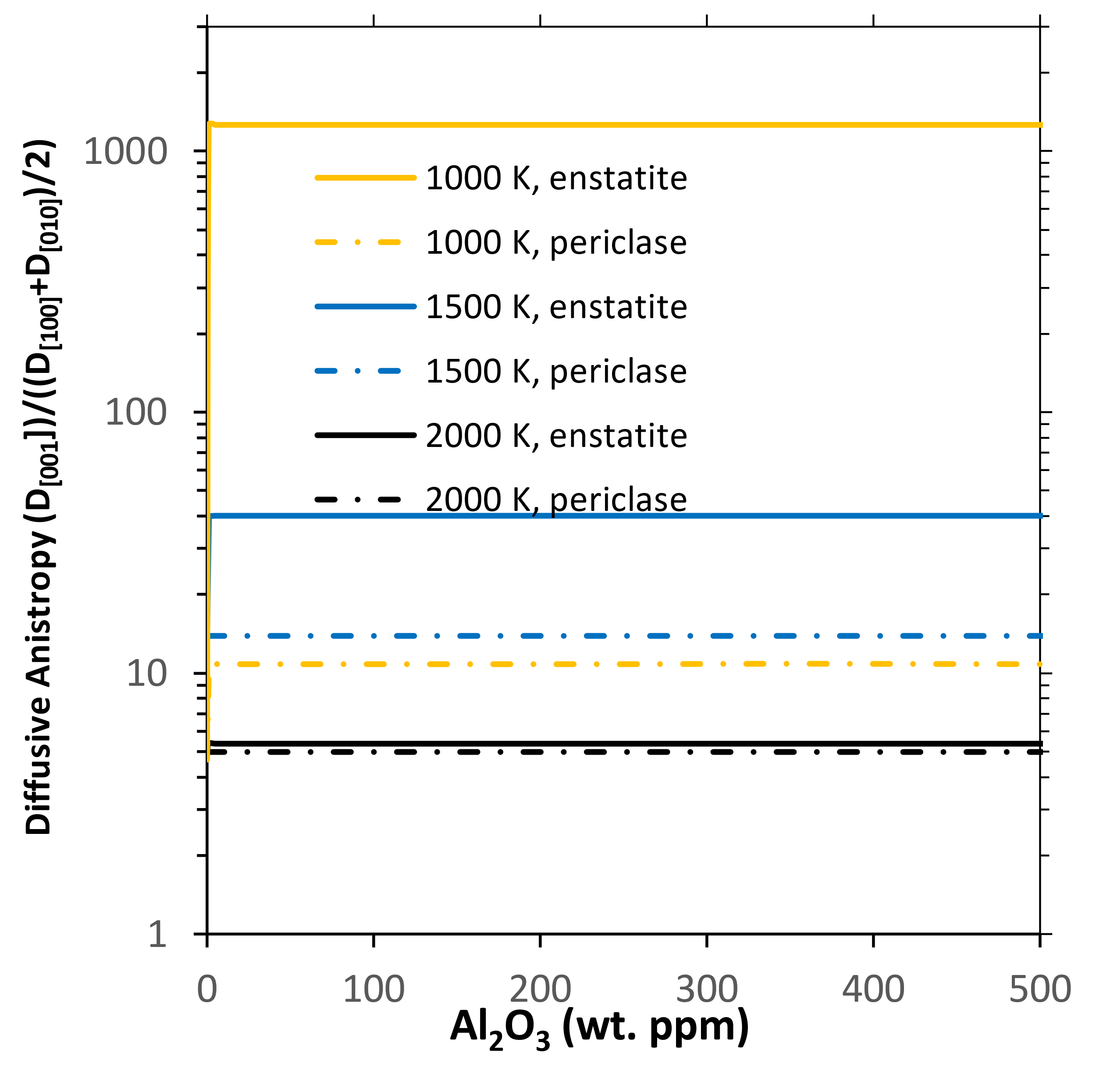


Fig S6- As Figure 6 but at 5 Gpa.


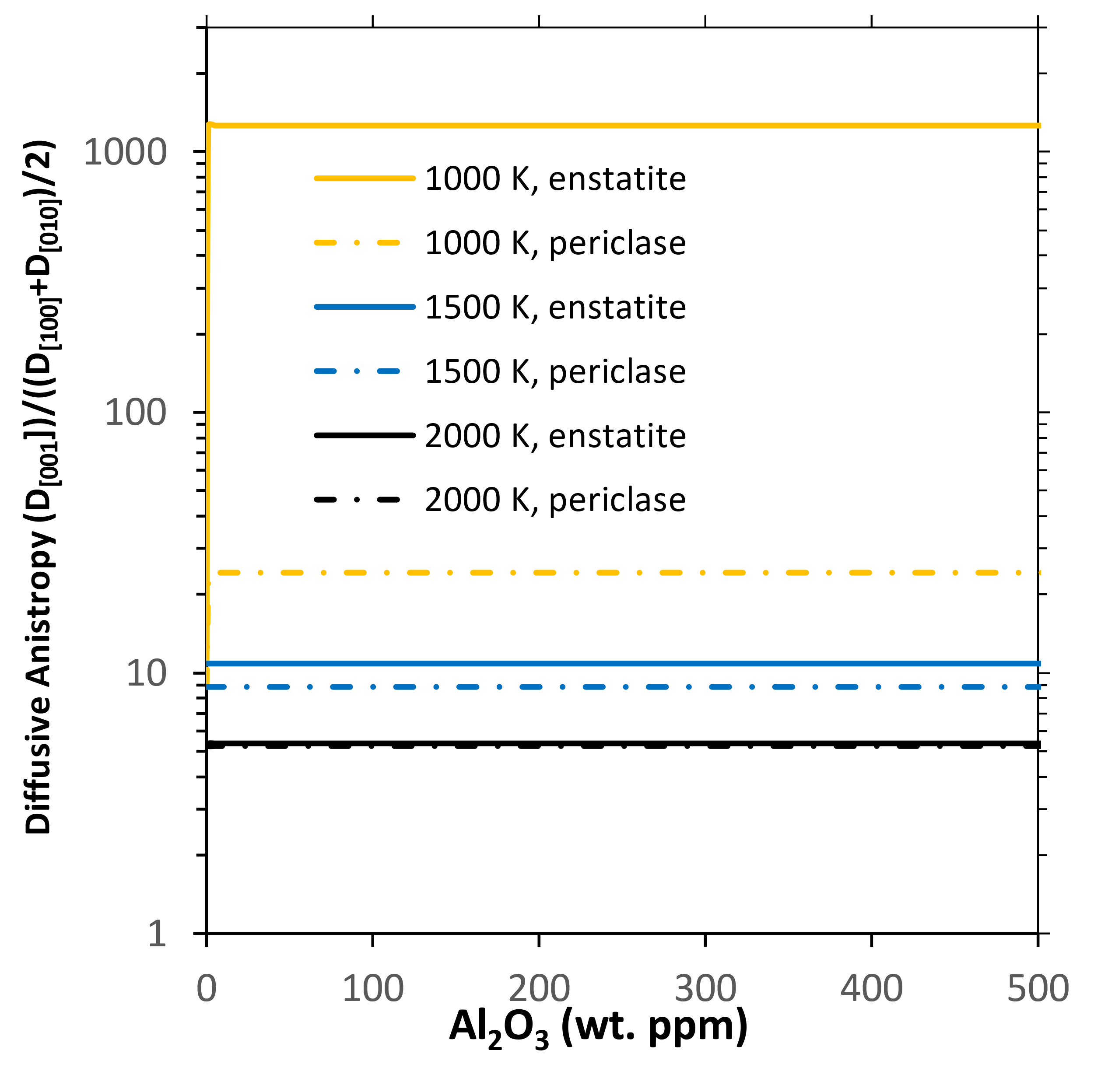


Fig S7- As Figure 6 but at 10 GPa


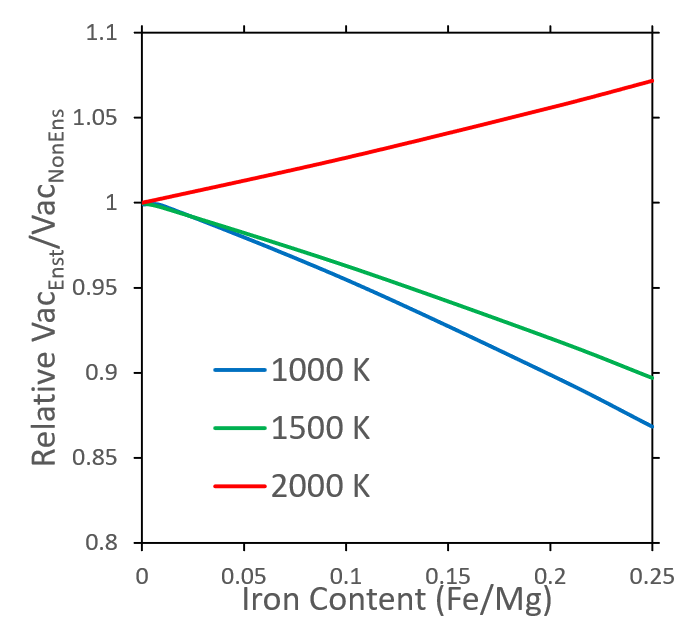


Figure S8 Effect of iron on the increase in vacancies caused by aluminium and enstatite for 50 ppm wt. Al_2_O_3_ at 0 GPa and various temperatures (colour).


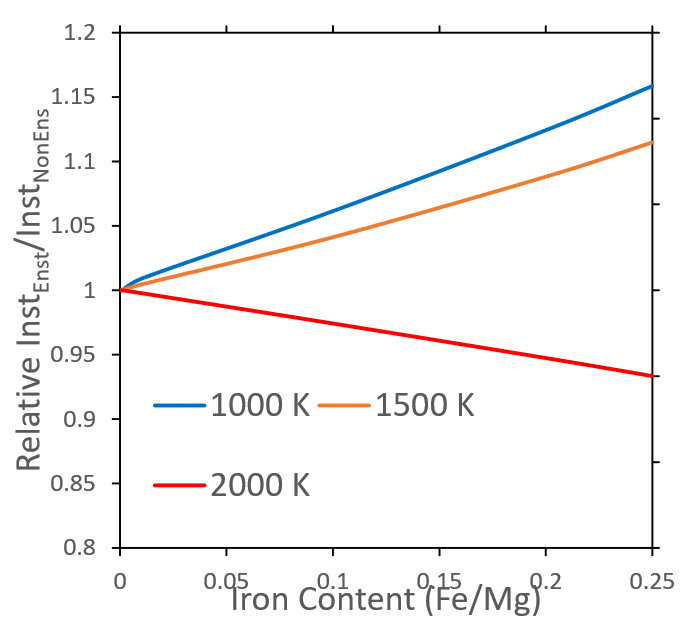


Figure S9- As Fig S8 but with interstitial vacancy concentrations.

|  | Energy at 0 K | Energy at 1000 K | Energy at 1500 K | Energy at 2000 K | Min Conc at 1500 K |
| --- | --- | --- | --- | --- | --- |
| $V_{Mg}^{''}+{Mg}_{I}^{\bullet\bullet}$ | -1.87 | -1.85 | -1.74 | -1.37 | 1.9 X 10^-6^ |
| ${Al}_{Mg}^{\bullet}+{Al}_{Si}^{'}$ | -0.98 | -1.03 | -1.06 | -1.11 | 6.6 X 10^-5^ |
| ${Al}_{Mg}^{\bullet}+V_{Mg}^{''}+{Al}_{Mg}^{\bullet}$ | -1.96 | -2.08 | -2.07 | -2.15 | 7.3 X 10^-5^ |

Table S1: Pairing Energies of some major defect pairs at 0 GPa and 0 K and high temperatures alongside the minimum concentration (in defects per formula unit) at 1500 K needed before pairing energy starts to dominate over configurational entropy. The pairing energies are relatively insensitive to temperature at this pressure. These numbers are much higher than the concentrations of defects we produce and thus all defect shall be considered as individual defects and not clusters.

|  |  | 0 K | 1000 | 1500 | 2000 |
| --- | --- | --- | --- | --- | --- |
| R11) Al+Enstatite Rection | 0 | 3.27 | 3.71 | 3.74 | 3.66 |
|  | 5 | 3.37 | 4.24 | 4.52 | 4.67 |
|  | 10 | 3.46 | 4.51 | 5.07 | 5.44 |
| R12) Ferric Iron+Enstatite  Reaction | 0 | 2.14 | 1.29 | 0.78 | 0.27 |
|  | 5 | 2.21 | 1.44 | 0.95 | 0.41 |
|  | 10 | 2.41 | 1.75 | 1.29 | 0.75 |
| R13) Al Without Enstatite | 0 | 5.24 | 5.83 | 5.96 | 6.01 |
|  | 5 | 4.93 | 6.13 | 6.69 | 7.21 |
|  | 10 | 4.76 | 6.43 | 7.21 | 7.74 |

Table S2: Energy (in eV) of R11 alongside its ferric iron equivalent (R12) and a version that does not use enstatite but produces MgO instead. Ferric iron reaction energies were calculated the same as Al reaction energies but ferric iron was set to high spin at the A site and B site and a DFT+U method was used with U_eff_ set to 3 eV.

|  |  | 0 ppm | 0.1 | 1 | 10 | 100 | 1000 |
| --- | --- | --- | --- | --- | --- | --- | --- |
| Relative Change in Mg Vacancy Concentration | 1000 K | 324947 | 1.02 | 1.00 | 1.00 | 1.00 | 1.00 |
|  | 1500 | 1453 | 19 | 2.82 | 1.18 | 1.00 | 1.00 |
|  | 2000 | 28 | 26 | 15 | 3.03 | 1.20 | 1.02 |
| Relative Change in Mg Interstitial Concentration | 1000 K | 0.02 | 0.02 | 0.02 | 0.02 | 0.04 | 0.77 |
|  | 1500 | 0.00 | 0.05 | 0.32 | 0.90 | 0.98 | 1.00 |
|  | 2000 | 0.01 | 0.01 | 0.02 | 0.09 | 1.00 | 1.00 |

Table S3: Relative Change in Mg vacancy and interstitial concentration upon adding enstatite to a forsterite crystal containing 50 wt. ppm Al as a function of background Mg vacancies (in defects/unit cell) at 0 GPa. With increasing Mg vacancies the effect of the enstatite hugely reduces. A value of 1 represents that enstatite causes no change to the concentration of defects. The concentration of background vacancies can be produced by many sources. For example they could be produced b y water where they can then be represented by Equation 13 and a similar equation could be derived for any vacancy producing substance. To convert to wt. ppm water multiply these values by ~1.282 if $\gamma=1$ in Equation 13 and then scale by $\gamma$.

|  | | 0 GPa | 5 | 10 |
| --- | --- | --- | --- | --- |
| $V_{Mg}^{''}$ | M1 | 0.000 | 0.000 | 0.000 |
|  | M2 | 1.117 | 1.199 | 1.250 |
| ${Mg}_{I}^{\bullet\bullet}$ | M1 | 0.000 | 0.000 | 0.000 |
|  | I2 | -0.004 | -0.155 | -0.237 |
|  | I2 1000 K | 0.315 | 0.209 | 0.192 |
|  | I2 2000 K | 0.468 | 0.372 | 0.378 |
|  | I2 1000 K | 0.623 | 0.524 | 0.543 |
| ${Al}_{Mg}^{\bullet}$ | M1 | 0.000 | 0.000 | 0.000 |
|  | M2 | 0.012 | -0.055 | -0.107 |
| $V_{O}^{\bullet\bullet}$ | O1 | 2.269 | 2.327 | 2.367 |
|  | O2 | 0.806 | 0.731 | 0.655 |
|  | O3 | 0.000 | 0.000 | 0.000 |
| $O_{I}^{''}$ | I1 | 1.203 | 1.180 | 1.156 |
|  | I2 | 0.015 | 0.016 | 0.016 |
|  | T1 | 1.487 | 1.542 | 1.609 |
|  | T2 | 0.000 | 0.000 | 0.000 |
|  | T3 | 0.015 | 0.017 | 0.015 |
|  | T4 | 0.741 | 0.889 | 1.027 |
|  | T5 | 0.017 | 0.018 | 0.016 |
| ${Si}_{I}^{\bullet\bullet\bullet\bullet}$ | I1 | 4.130 | 4.122 | 4.114 |
|  | I2 | 1.927 | 1.821 | 1.978 |
|  | T1 | 0.279 | 0.000 | 0.000 |
|  | T2 | 1.660 | 1.519 | 1.641 |
|  | T3 | 0.000 | 0.149 | 0.530 |
|  | T4 | 0.000 | 0.150 | 1.654 |
|  | T5 | 3.010 | 3.246 | 3.482 |

Table S4: Relative enthalpy (in eV) of defects in various sites- at each pressure enthalpies are set relative to the lowest energy defect site as are the reaction energies. For all defects we used these static enthalpy values to represent the difference in energy between sites except for Mg interstitials. Due to the large difference in geometry between interstitials located on M1 and I2 sites their phonons are quite different and this leads to a strong temperature dependence for the energy of placing a defect at each site with higher temperatures strongly favouring M1 sites. The high temperature relative energies are listed here and these were included in the model. We tested the temperature dependence of relative site energy for two other prominent defects- Mg vacancies and Aluminium on the Mg site- for M1 and M2 sites but found including the temperature changed the relative site energy by <10 meV/defect and thus was not included in the model. This is because the phonons of these defects are near identical in similar sites and it is likely to be the same for all defects unless there is substantial rearrangement of other non-defect atoms as occurs when you go from an Mg interstitial sitting on the I2 site to a split interstitial M1 arrangement which moves another non-defect Mg atom.

|  |  | 0 K | 1000 | 1500 | 2000 |
| --- | --- | --- | --- | --- | --- |
| R2) O Frenkel | 0 | 10.02 | 10.27 | 10.53 | 10.92 |
|  | 5 | 10.50 | 11.16 | 11.61 | 12.18 |
|  | 10 | 10.92 | 11.93 | 12.56 | 13.29 |
| R3) Si Frenkel | 0 | 26.92 | 25.76 | 26.19 | 27.54 |
|  | 5 | 34.93 | 32.59 | 32.21 | 32.59 |
|  | 10 | 43.77 | 40.44 | 39.36 | 38.87 |
| R5) Mg-O Interstitial | 0 | 8.98 | 8.81 | 8.86 | 9.06 |
|  | 5 | 8.86 | 9.02 | 9.22 | 9.55 |
|  | 10 | 8.79 | 9.26 | 9.59 | 10.03 |
| R6) Mg Vac Si Int | 0 | 21.09 | 20.83 | 21.60 | 23.19 |
|  | 5 | 28.88 | 27.86 | 28.03 | 28.84 |
|  | 10 | 37.58 | 35.97 | 35.65 | 35.82 |
| R7) Si-O Vac | 0 | 20.58 | 19.57 | 19.06 | 18.61 |
|  | 5 | 22.04 | 21.38 | 20.95 | 20.52 |
|  | 10 | 23.31 | 23.10 | 22.85 | 22.51 |
| R8) Si Vac  Mg Int | 0 | 18.51 | 17.14 | 15.97 | 15.08 |
|  | 5 | 19.06 | 17.93 | 16.78 | 15.81 |
|  | 10 | 19.51 | 18.24 | 17.39 | 16.47 |
| R9) Schottky | 0 | 34.23 | 36.44 | 37.25 | 39.31 |
|  | 5 | 37.05 | 39.54 | 41.55 | 43.76 |
|  | 10 | 39.56 | 43.30 | 45.82 | 48.40 |

Table S5: Energy (in eV) of the less favourable reactions as a function of pressure and temperature without considering configurational entropy.
